# Supplementary material for: Performance of tumour microenvironment deconvolution methods in breast cancer using single-cell simulated bulk mixtures
Source: Nat Commun. 2023 Sep 16;14:5758. doi: 10.1038/s41467-023-41385-5 (PMC10505141; doi:10.1038/s41467-023-41385-5)
Supplement: Supplementary file 1 — Supplementary Information [file 41467_2023_41385_MOESM1_ESM.pdf]

Performance of tumour microenvironment deconvolution methods in breast cancer using single-cell simulated bulk mixtures

Supplementary Information

Khoa A. Tran[1,2], Venkateswar Addala[1], Rebecca L. Johnston[1], David Lovell[3,4], Andrew Bradley[5], Lambros T. Koufariotis[1], Scott Wood[1], Sunny Z. Wu[6,7], Dan Roden[6,7], Ghamdan Al-Eryani[6,7], Alexander Swarbrick[6,7], Elizabeth D. Williams[2,8], John V. Pearson[1], Olga Kondrashova[1*], Nicola Waddell[1,2*#]

*Jointly supervised and contributed to the work

[1] Medical Genomics and Genome Informatics, QIMR Berghofer Medical Research Institute, Brisbane, 4006, Australia

[2] School of Biomedical Sciences at the Translational Research Institute (TRI), Queensland University of Technology (QUT), Brisbane, 4000, Australia

[3] School of Computer Science, Queensland University of Technology, Brisbane, 4000, Australia

[4] QUT Centre for Data Science, Brisbane, 4000, Australia

[5] Faculty of Engineering, Queensland University of Technology, Brisbane, 4000, Australia

[6] Cancer Ecosystems Program, Garvan Institute of Medical Research, Darlinghurst, NSW 2010, Australia

[7] School of Clinical Medicine, Faculty of Medicine and Health, UNSW Sydney, Kensington, NSW 2052, Australia

[8] Australian Prostate Cancer Research Centre – Queensland (APCRC-Q) and Queensland Bladder Cancer Initiative (QBCI), Brisbane, 4000, Australia

# Corresponding author: Dr Nicola Waddell, Cancer Program, QIMR Berghofer Medical Research Institute, 300 Herston Road, Brisbane, 4006, Australia. Tel: +61 7 3845 3538. email: Nic.waddell@qimrberghofer.edu.au. ORCID: 0000-0002-3950-2476

Supplementary Note 1

Technical overviews of deconvolution methods

BayesPrism

BayesPrism[1] is a Bayesian method which uses single-cell reference profiles as the prior to estimate a joint posterior of cell-type fractions and cell-type-specific gene expressions. With each bulk mixture, the algorithm updates the posterior distribution using Gibbs sampling[2]. This process is executed for each cell state, i.e. cell subtype, present in the single-cell reference, then cell-state fractions and gene expressions are summed over all cell states of each cell type. Furthermore, BayesPrism also factors in the presence of cancer cells and splits the single-cell reference into a malignant reference (used for deconvolving cancer population), and a non-malignant reference (used for deconvolving non-cancer populations).

bisqueRNA (Bisque)

Bisque[3] is designed specifically to address issues that may originate from the proportionality assumptions between single-cell reference and bulk mixtures expression values. That is, many regression-based deconvolution methods rely on the assumption that single-cell reference and bulk mixture expression values are from the same distributions. However, this is not always true, as the methods that generate bulk and single-cell sequencing data, such as library preparation and mRNA capture methods, can differ greatly. Bisque overcomes these potential biases by transforming gene-specific bulk expression values into the same distribution space with gene-specific expression values of the single-cell reference data. Bisque achieves this by first averaging gene counts within each cell type across the single-cell reference matrix and then multiplying the results with cell type proportions of each patient (normalised counts of cells within each cell type), producing a “pseudo-bulk” reference sample for each individual. If single-cell and bulk expression values come from the same patient, expression of each gene in bulk mixtures will be transformed to the same distribution space as the “pseudo-bulk” reference via linear regression.

CIBERSORTx (CBX)

CBX[4] is an improvement of an earlier method called CIBERSORT[5] (CB). Both CB and CBX use the same linear Support Vector Regression (SVR) algorithm to impute cell fractions using the same formula as other regression methods:

$$(1)\, B * F_i = M_i$$

where B is a signature matrix containing *n* discriminatory marker genes across *c* cell types, F_i is fractions of *c* cell types in mixture j, and M_i is bulk expression of n genes in bulk mixture. While CB relies on the signature matrix LM22[5], CBX has built-in functionality to build a custom signature matrix using any scRNA-seq dataset. Essentially, this method filters out lowly expressed genes, i.e. those with transcripts per million (TPM) < 0.75 in $\log_2$ scale, randomly selects 50% of available expression profiles for each cell type with count > 3, then sums their gene expression values and normalises gene expression values in non-log space into TPM. This process is repeated five times (default setting of CBX), with gene expression values averaged across duplicates to produce a final signature matrix.

Cell Population Mapping (CPM)

CPM[6] performs deconvolution following a sequence of steps that traverse through a cell state space that captures the essence of gene-regulation variation among the reference single cells. This cell state space can be generated using dimensionality reduction techniques such as tSNE or UMAP.

By integrating the cell state space and reference single-cell expression values, CPM performs deconvolution by repeating many iterations of:

1) randomly sampling N number of reference subsets (each containing Ns cells) from the provided reference single cell (N is decided so that each single cell is randomly sampled at least Nr times),
2) predicting abundance of each cell in the Ns subset using the same linear SVR model in CBX[4] and its earlier version CIBERSORT[5],

3) averaging the abundance of each individual single cell over N runs (as one single cell can be sampled for a Ns subset at least Nr times)
4) computing smoothed abundance of each individual single cell as the averaged abundance of its Nd nearest-neighbour cell. This is to generate a smooth distribution of cell abundance across the entire cell space,
5) estimating cell-type-level abundance by averaging single-cell abundances across cell types.

Dampened Weighted Least Squares (DWLS)

DWLS[7] relies on a single cell reference matrix to build its internal signature matrix in a 3-step process. First, DWLS clusters cells in the single-cell reference into different cell types using a rare-cell-type-sensitive clustering method and the provided unique cell types. After that, different expression analyses are conducted between cell clusters (using the MAST library) to detect distinct clusters containing biologically relevant cell types and identify marker genes for each cell type. DWLS defines marker genes as those with False Discovery Rate (FDR) adjusted *p*-value < *0.01* and log_2 mean fold change > 0.5. Next, 151 candidate signature matrices are created by selecting between 50-200 (hence 151) marker genes from each cell type. Within each candidate matrix, gene expression values are averaged across cell types. Similar to CIBERSORT, the candidate matrix with the lowest condition number is chosen.

DWLS then performs an improved version of the Ordinary Least Squares (OLS) method to deconvolute cell proportions. Traditional OLS methods model bulk expressions (t) as a function of signature gene expression values (S) and proportions of cells in the bulk mixture (x) in a linear relationship:

$$(2)\ t = S * x$$

By iterating over the provided data, DWLS (and any least-squares-based models) attempts to produce a set of x that minimizes the error function:

$$(3)\ Error = t - B * x$$

This function can be refactored as a multiplication of relative percent error, *S* and *x*. This leads to two limitations of traditional OLS: 1) rare cell types with very small x have little to no impact on the error function, and 2) genes pertaining to prevalent cell types usually dominate the error term, regardless of whether they are differentially expressed. The second limitation also implies that differentially expressed genes with low expression play very minimal roles in the error terms. To address these two issues, DWLS introduces a weight to "weight down" the impact of abundant cell types such that

$$(4)\ Error = W * (t - B * x)$$

Estimating the Proportion of Immune and Cancer cells (EPIC)

EPIC[8] relies on gene markers for the cell types it can deconvolve to estimate populations of these cell types. The rest of the cell population is characterised as uncharacterized cell types, where the expression of signature genes is either very low or non-existent. This uncharacterised cell population can be referred to as cancer population.

hybrid-scale proportions estimation (hspe)

hspe[9] is the updated version of dtangle[10] benchmarked in Cobos et al[11]. The inspiration for dtangle and hspe is that most existing deconvolution methods are regression-based models which require gene expression values either in linear or logarithmic scales. Both dtangle and hspe assert that while log-scale gene expression values are biologically questionable, linear-scale gene expression values have unrealistic error assumptions. Both methods rely on the use of a hybrid linear-logarithm approach to overcome these limitations. In our benchmarking study, hspe and dtangle produced identical results. We, therefore, report only results of hspe representative of both methods.

MUlti-Subject SIngle Cell deconvolution (MuSiC)

Similar to other single-cell-based methods benchmarked in this study, MuSiC[12] uses single-cell reference profiles to build its own set of marker genes. The method, however, implemented an innovation in its marker gene selection algorithm and another innovation in its quantification of cell-type abundance. The first innovation comes from its multi-subject gene-weighting approach. MuSiC dynamically emphasizes the importance of genes with low cross-subject variances by increasing their weights compared to the weights of genes with low cross-subject variances. This technique essentially incorporates cell-type-specific gene expression into the method's algorithm. MuSiC's second innovation was to deal with high collinearity of gene expression values among closely related cell types by incorporating a hierarchical deconvolution structure. The algorithm first groups cell types into clusters and estimate cluster-specific proportions. Within each cluster, MuSiC recursively repeats the same process to arrive at cell-type-specific proportions.

Single cell–assisted deconvolutional DNN (Scaden)

Scaden[13] is an artificial intelligence method that uses an ensemble of three deep neural networks to infer the cellular composition of bulk tissues. By using gene expression values of simulated bulk mixtures to predict cell compositions, the three neural networks in Scaden "learn" how to adjust the weight of each neuron to get their predictions as close to the ground truth as possible. Each of the three neural networks in Scaden produce their own set of predictions, which are averaged to produce the method's final prediction.

Supplementary Note 2

Library size of simulated bulk mixtures

The recommended total RNA input for bulk RNA-seq library preparation can be as low as 10ng (SMART-seq) or 25ng (Illumina Stranded mRNA prep), which translates to 500 or 1,250 cells given the assumption that a typical mammalian cell contains 10-30 pg of total RNA. We chose to use 500 cells per pseudobulk to ensure we had enough cells for the simulated mixtures, particularly for the cancer purity experiments (where cancer cells made up to 95% of the mixture). As some samples contained between 500-1,000 cells for the most prevalent cell type that was used as the cell number for SMOTE oversampling, we were unable to SMOTE more cells than this number. We wanted to avoid sampling some cells twice, which would have been required if we used a higher number of cells per pseudo-bulk.

In terms of the library size, the average number of reads per cell is around 7,000 in Wu et al[14]. This adds up to around 3.5 million reads per pseudobulk mixture, which is less than 25-50 million reads for a typical real bulk mixture. We did, however, normalise scRNA-seq read counts to counts-per-10,000, which was comparable to using transcripts-per million normalised read counts in real bulk. We note that the smaller total library size means genes with lower expression would be less represented compared to bulk RNA-seq, which is a potential limitation of our study design. However, due to cell number constraints outlined above, we are unable to adjust the design.

Supplementary Figures

Supplementary Fig. 1: Number of cells per cell type before and after data oversampling using SMOTE in each breast cancer patient.
Supplementary Fig. 2: UMAP visualisations of synthesised and original cells from 26 breast cancer patients.
Supplementary Fig. 3: Cell counts for nine major cell types across training simulated variable tumour purity mixtures.
Supplementary Fig. 4: Impact of variable tumour purity levels on deconvolution for nine methods.
Supplementary Fig. 5: Performance of CPM across tumour purity levels.
Supplementary Fig. 6: Direction of mis-predictions of each cell type for nine computational methods.
Supplementary Fig. 7: Overall generalisation of impact of tumour purity on deconvolution to simulated bulk mixtures generated using scRNA-Seq from Bassez et al and Pal et al.
Supplementary Fig. 8: Cell-type-specific generalisation of impact of tumour purity on deconvolution to simulated bulk mixtures generated using scRNA-Seq from Bassez et al and Pal et al.
Supplementary Fig. 9: Histogram of predicted cancer proportions.
Supplementary Fig. 10: Studying the impact of tumour purity on deconvolution to simulated bulk mixtures generated using scRNA-Seq from Bassez et al and Pal et al, and single-cell reference from Wu et al.
Supplementary Fig. 11: Cell-type specific deconvolution performance for nine methods with tumour and normal epithelial cells grouped as epithelial.
Supplementary Fig. 12: The performance of the nine deconvolution methods assessed by false positive and false negative rates.
Supplementary Fig. 13: Cross-lineage performance of BayesPrism and DWLS by patient.
Supplementary Fig. 14: Raw prediction errors of BayesPrism and DWLS across immune lineages.
Supplementary Fig. 15: The performance of BayesPrism and DWLS methods for minor and subset immune cell types assessed by false positive and false negative rates.
Supplementary Fig. 16: Comparison of nine deconvolution approaches to four alternative methods to predict tumour purity in TCGA breast data.
Supplementary Fig. 17: Performance of BayesPrism before and after Gibbs sampling on artificial bulk mixtures generated using scRNA-seq data from Wu et al, Bassez et al and Pal et al.
Supplementary Fig. 18: Performance comparison of different versions of BayesPrism, Bisque, MuSiC, hspe and DWLS.

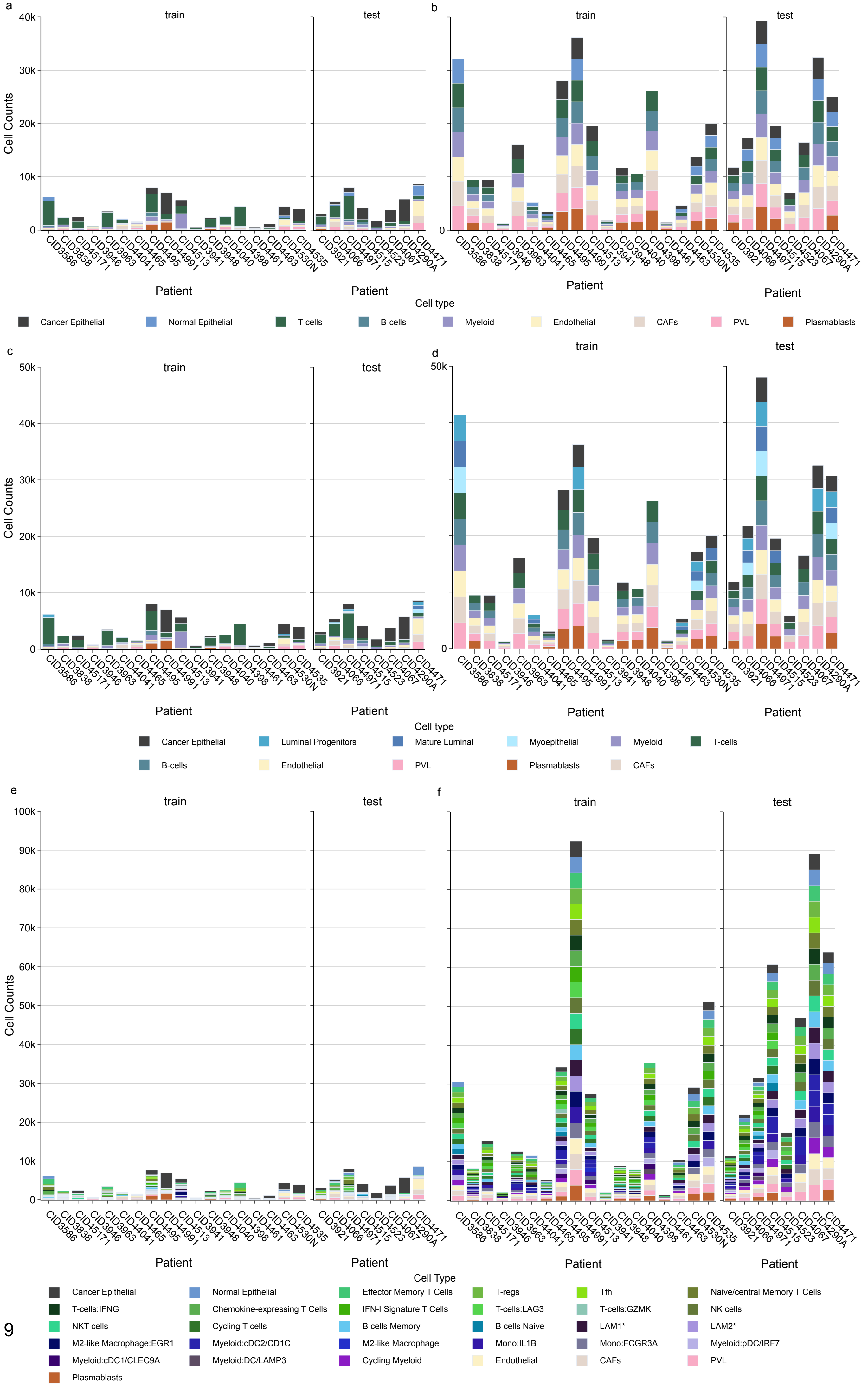

a
b
c
d
e
f
train
test
Cell Counts
Patient
CID3586
CID3838
CID45171
CID3946
CID3963
CID44041
CID4465
CID4495
CID44991
CID4513
CID3941
CID3948
CID4040
CID4398
CID4461
CID4463
CID4530N
CID4535
CID3921
CID4066
CID44971
CID4515
CID4523
CID4067
CID4290A
CID4471
Cell type
Cancer Epithelial
Normal Epithelial
T-cells
B-cells
Myeloid
Endothelial
CAFs
PVL
Plasmablasts
Luminal Progenitors
Mature Luminal
Myoepithelial
Cell Type
Effector Memory T Cells
T-regs
Tfh
Naive/central Memory T Cells
T-cells:IFNG
Chemokine-expressing T Cells
IFN-I Signature T Cells
T-cells:LAG3
T-cells:GZMK
NK cells
NKT cells
Cycling T-cells
B cells Memory
B cells Naive
LAM1*
LAM2*
M2-like Macrophage:EGR1
Myeloid:cDC2/CD1C
M2-like Macrophage
Mono:IL1B
Mono:FCGR3A
Myeloid:pDC/IRF7
Myeloid:cDC1/CLEC9A
Myeloid:DC/LAMP3
Cycling Myeloid
9

Supplementary Fig. 1: Number of cells per cell type before and after data oversampling using SMOTE in each breast cancer patient. Number of cells for each of the nine major cell types per patient before (**a**) and after data oversampling used to assess impact of tumour purity levels on deconvolution (**b**)**.** Number of cells for each of the six non-normal-epithelial cell types and three minor normal epithelial cell types per patient before (**c**) and after data oversampling used to assess impact of normal cell lineages on deconvolution (**d**)**.** Number of cells for each of the five non-immune cell types, as well as 17 subset cell types and six minor cell types of T-cells, B-cells and Myeloid for each patient before **(e)** and after data oversampling used to assess impact of immune cell lineages on deconvolution (**f**)**.** All original cell counts before data oversampling were obtained from Wu *et al,* Nature Genetics 2021. Source data are provided as a Source Data file.

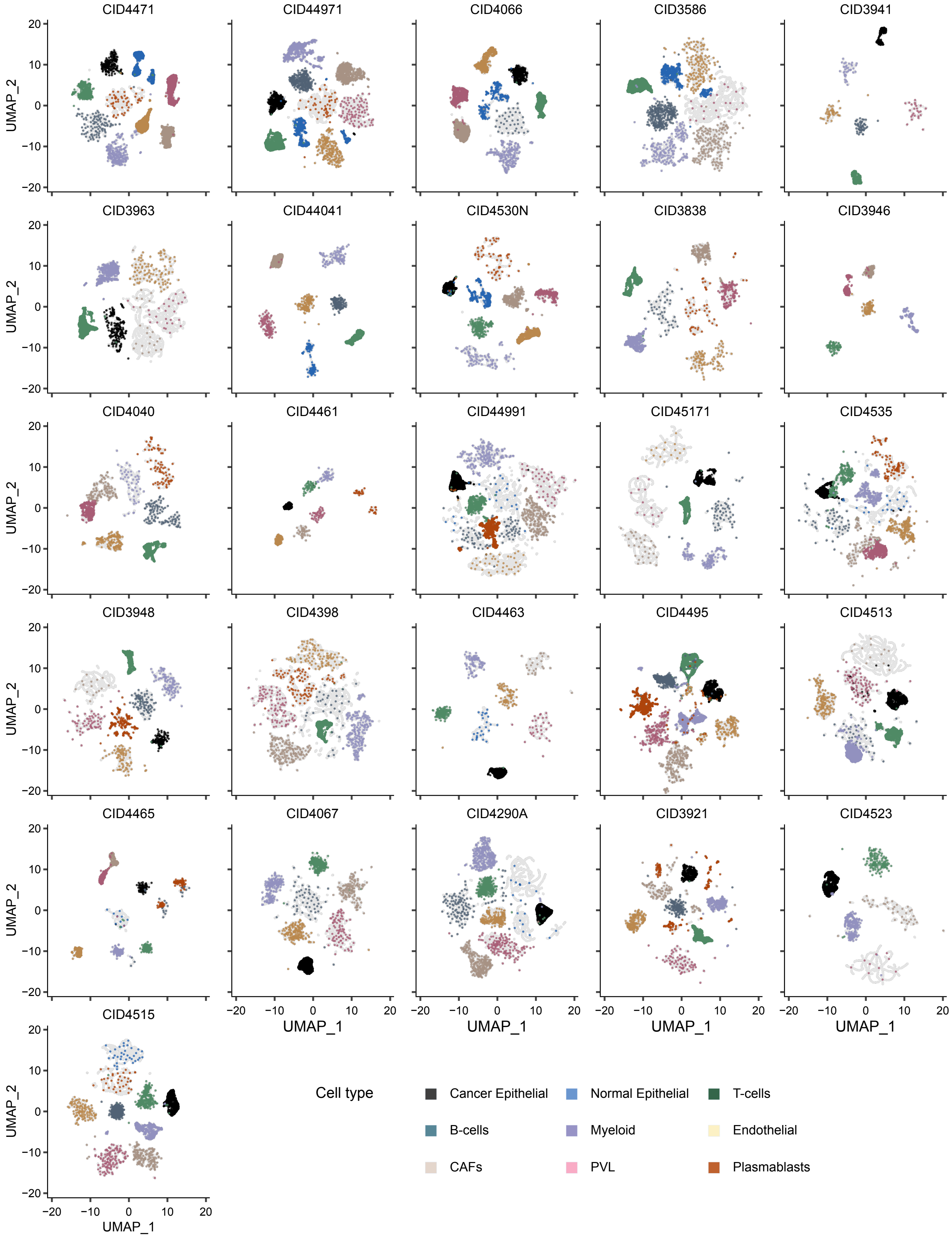
CID4471
CID44971
CID4066
CID3586
CID3941
CID3963
CID44041
CID4530N
CID3838
CID3946
CID4040
CID4461
CID44991
CID45171
CID4535
CID3948
CID4398
CID4463
CID4495
CID4513
CID4465
CID4067
CID4290A
CID3921
CID4523
CID4515
UMAP_1
UMAP_2
Cell type
Cancer Epithelial
Normal Epithelial
T-cells
B-cells
Myeloid
Endothelial
CAFs
PVL
Plasmablasts

Supplementary Fig. 2: UMAP visualisations of synthesised and original cells from 26 breast cancer patients. UMAP was run on gene expression values of both synthesised and original cells for each individual patient. Within each plot, grey points represent SMOTE synthesised cells across all cell types, and points of other colours represent original cells from each of the nine major cell types. Source data are provided as a Source Data file.

a

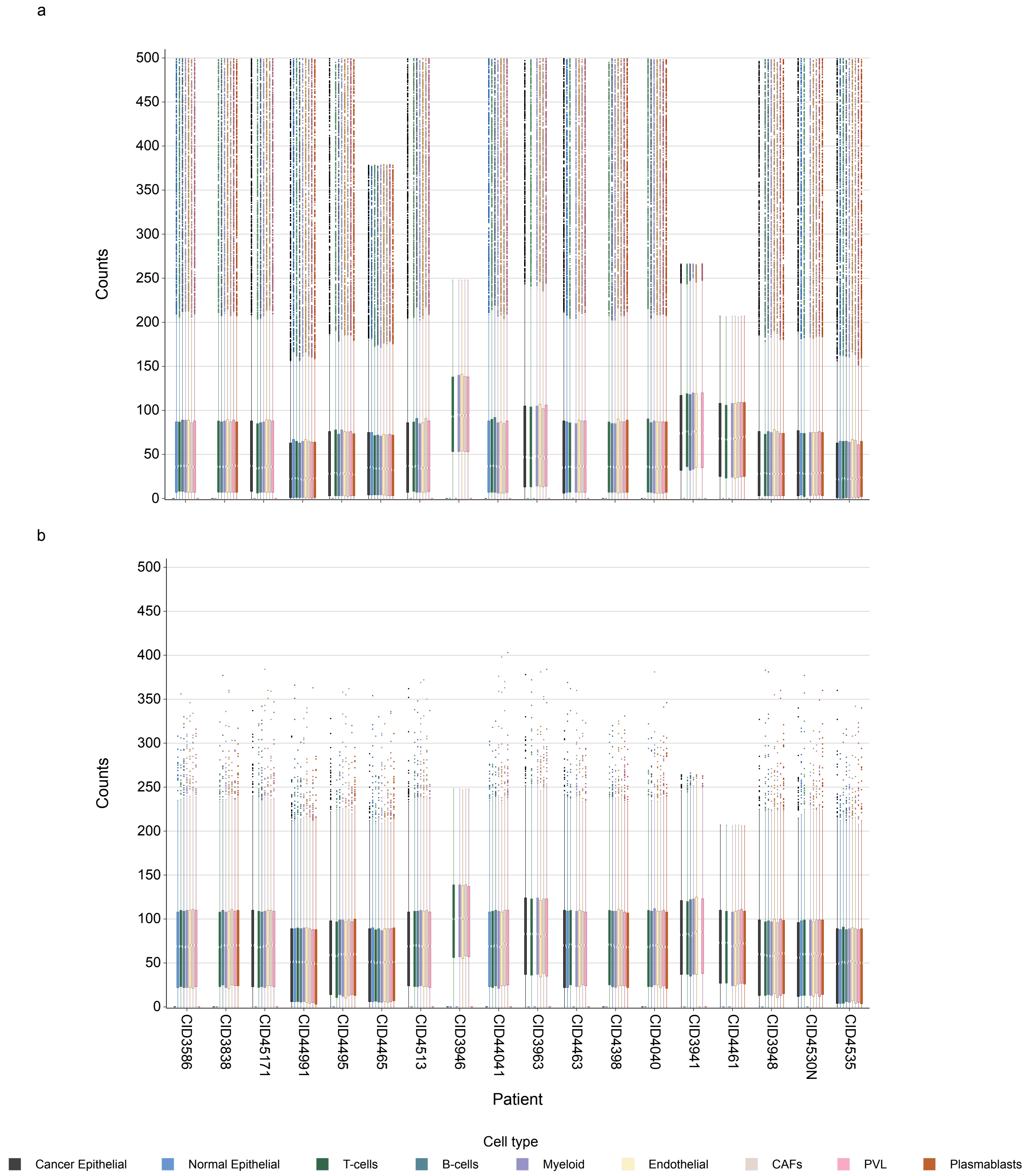

a
b
Counts
500
450
400
350
300
250
200
150
100
50
0
CID3586
CID3838
CID45171
CID44991
CID4495
CID4465
CID4513
CID3946
CID44041
CID3963
CID4463
CID4398
CID4040
CID3941
CID4461
CID3948
CID4530N
CID4535
Patient
Cell type
Cancer Epithelial
Normal Epithelial
T-cells
B-cells
Myeloid
Endothelial
CAFs
PVL
Plasmablasts

Supplementary Fig. 3: Cell counts for nine major cell types across training simulated variable tumour purity mixtures. The total of 38,000 mixtures across 19 tumour purity levels for each patient in the training dataset is shown (n=38,000 per boxplot). Boxplots represent cell counts in mixtures generated using the sparse method which was utilised in this study **(a)**, and cell counts in mixtures simulated using method from Menden *et al,* Science Advances 2020 **(b)**. All simulated mixtures had 500 cells, therefore a point closer to 500 on y-axis indicates a cell type accounting for the majority of a simulated mixture, while a point closer to 0 on the y-axis indicates a cell type with low representation in a simulated mixture. The sparse method enabled a more diverse proportion range across all cell types. Upper and lower whiskers depict cell counts outside of the centre 50%. Each box represents the middle 50% of cell-type counts per mixture, which includes the first quartile (Q1), the median, and the third quartile (Q3). Upper and lower whiskers depict maxima and minima of cell-type counts, excluding outliers. Outliers are cell-type counts that are more than 1.5x the interquartile range from either Q1 or Q3. Source data are provided as a Source Data file.

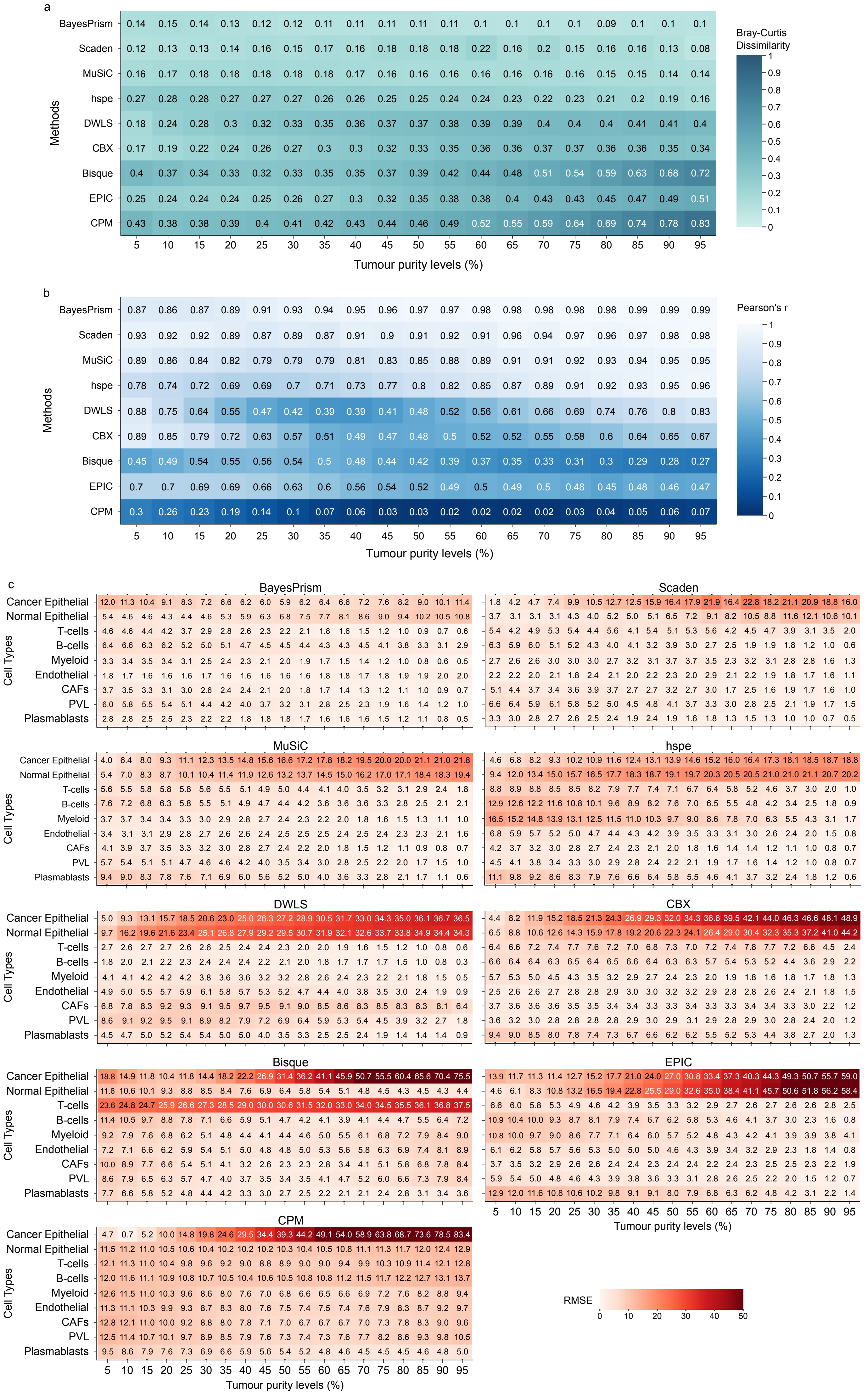

a
Methods
BayesPrism
Scaden
MuSiC
hspe
DWLS
CBX
Bisque
EPIC
CPM
Tumour purity levels (%)
Bray-Curtis Dissimilarity
b
Methods
Tumour purity levels (%)
Pearson's r
c
BayesPrism
Scaden
MuSiC
hspe
DWLS
CBX
Bisque
EPIC
CPM
Cell Types
Cancer Epithelial
Normal Epithelial
T-cells
B-cells
Myeloid
Endothelial
CAFs
PVL
Plasmablasts
Tumour purity levels (%)
RMSE

Supplementary Fig. 4: Impact of variable tumour purity levels on deconvolution for nine methods. a) Median Bray-Curtis dissimilarity and **b)** Median Pearson's r correlation coefficients between predicted and actual cell compositions across 19 tumours purity levels (samples with tumour cells ranging from 5% to 95%, at 5% intervals) which are shown as proportions (x-axis) for nine computational methods (y-axis). There were 2,000 artificial bulk mixtures per purity level. Darker shade of teal represents higher Bray-Curtis dissimilarity (worse performance), and darker shade of blue represents lower Pearson's r values (worse performance). The numeric values are shown. The computational methods are organised in order of decreasing performance according to their median Bray-Curtis dissimilarity values. Upper and lower whiskers depict Bray-Curtis values outside of the centre 50%. **c)** Median RMSE of each of the nine major cell types across all 2,000 artificial mixtures and 19 tumour purity levels (from 5% to 95%, 5% interval). Darker shades of red indicate higher RMSE values and worse deconvolution performance. The numeric RSME values are shown. Methods are ordered in the same order as **(a)**. RMSE: Root Mean Square Error. Source data are provided as a Source Data file.

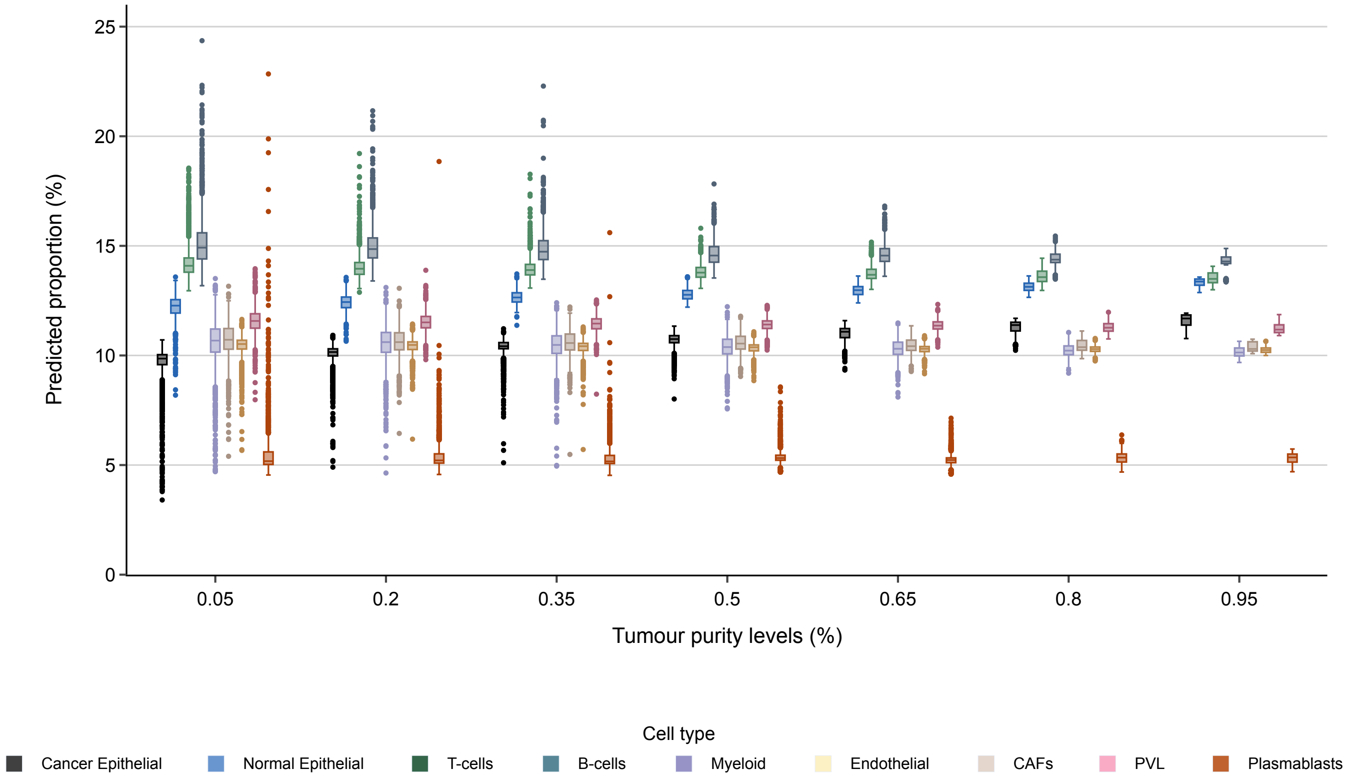

Supplementary Fig. 5: Performance of CPM across tumour purity levels. Predicted proportions of the CPM method across 7 tumour purity levels (from 5% to 95%, 15% interval). At each tumour purity level, proportions are aggregated into nine major cell types. Upper and lower whiskers depict predicted cell-type proportions outside of the centre 50%. Each box represents the middle 50% of predicted cell-type proportions, which includes the first quartile (Q1), the median, and the third quartile (Q3). Upper and lower whiskers depict maxima and minima of cell-type proportions, excluding outliers. Outliers are cell-type proportions that are more than 1.5x the interquartile range from either Q1 or Q3. Source data are provided as a Source Data file.

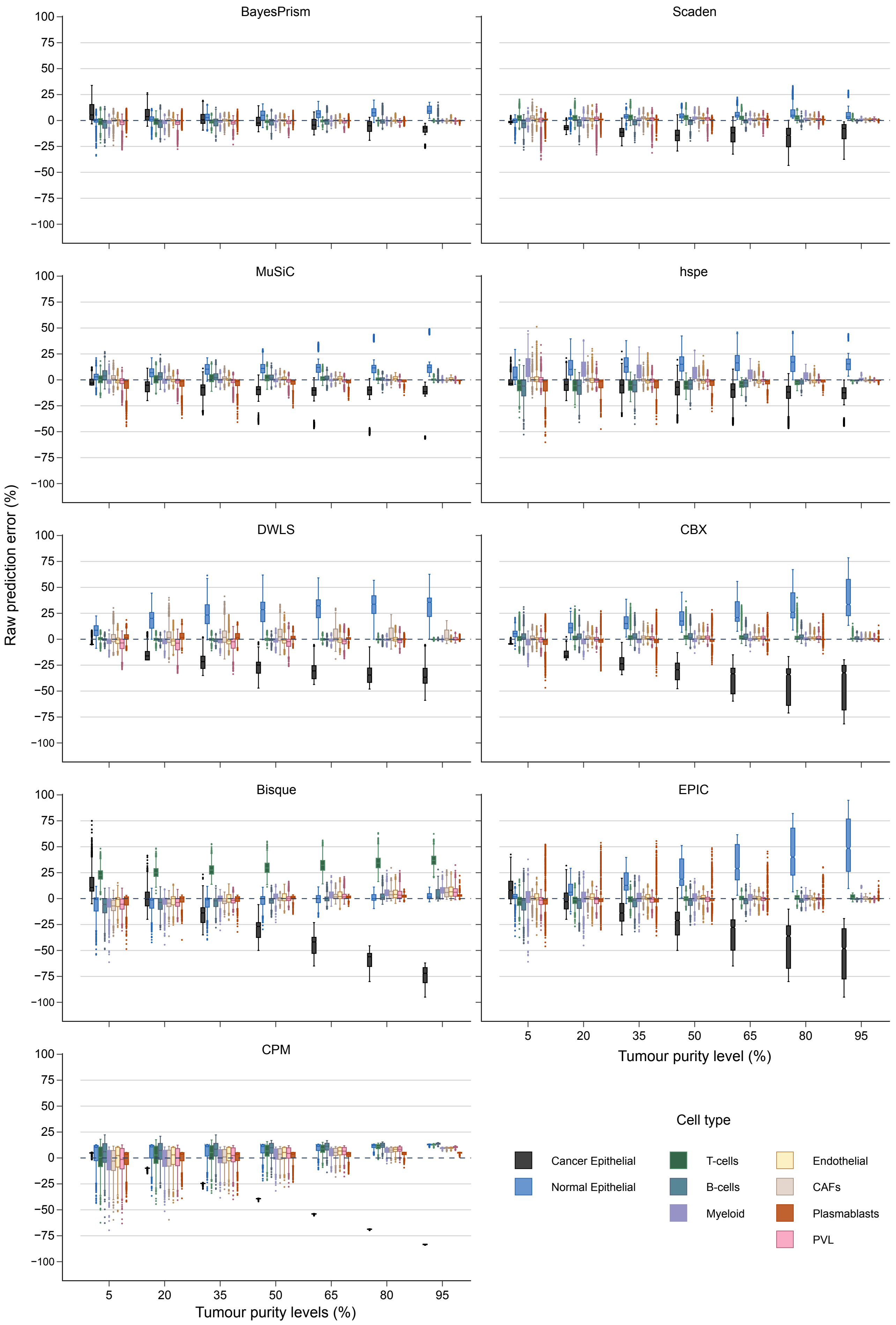

BayesPrism
Scaden
MuSiC
hspe
DWLS
CBX
Bisque
EPIC
CPM
Raw prediction error (%)
100
75
50
25
0
−25
−50
−75
−100
5
20
35
50
65
80
95
Tumour purity level (%)
Tumour purity levels (%)
Cell type
Cancer Epithelial
Normal Epithelial
T-cells
B-cells
Myeloid
Endothelial
CAFs
Plasmablasts
PVL

Supplementary Fig. 6: Direction of mis-predictions of each cell type for nine computational methods. Raw prediction errors (Predicted – Actual Cell Fractions) between predicted and ground truth cell compositions of nine major cell types across 7 tumour purity levels (from 5% to 95%, 15% interval). Higher positive and lower negative raw prediction errors represent worse performance. Each of the nine plots represents raw prediction errors for one deconvolution method. Negative raw prediction errors represent under-estimation (predicted fraction lower than ground truth), positive raw prediction errors represent over-estimation (prediction fraction higher than ground truth). Upper and lower whiskers depict raw prediction errors outside of the centre 50%. Each box represents the middle 50% of raw prediction errors, which includes the first quartile (Q1), the median, and the third quartile (Q3). Upper and lower whiskers depict maxima and minima of raw prediction errors, excluding outliers. Outliers are raw prediction errors that are more than 1.5x the interquartile range from either Q1 or Q3. Source data are provided as a Source Data file.

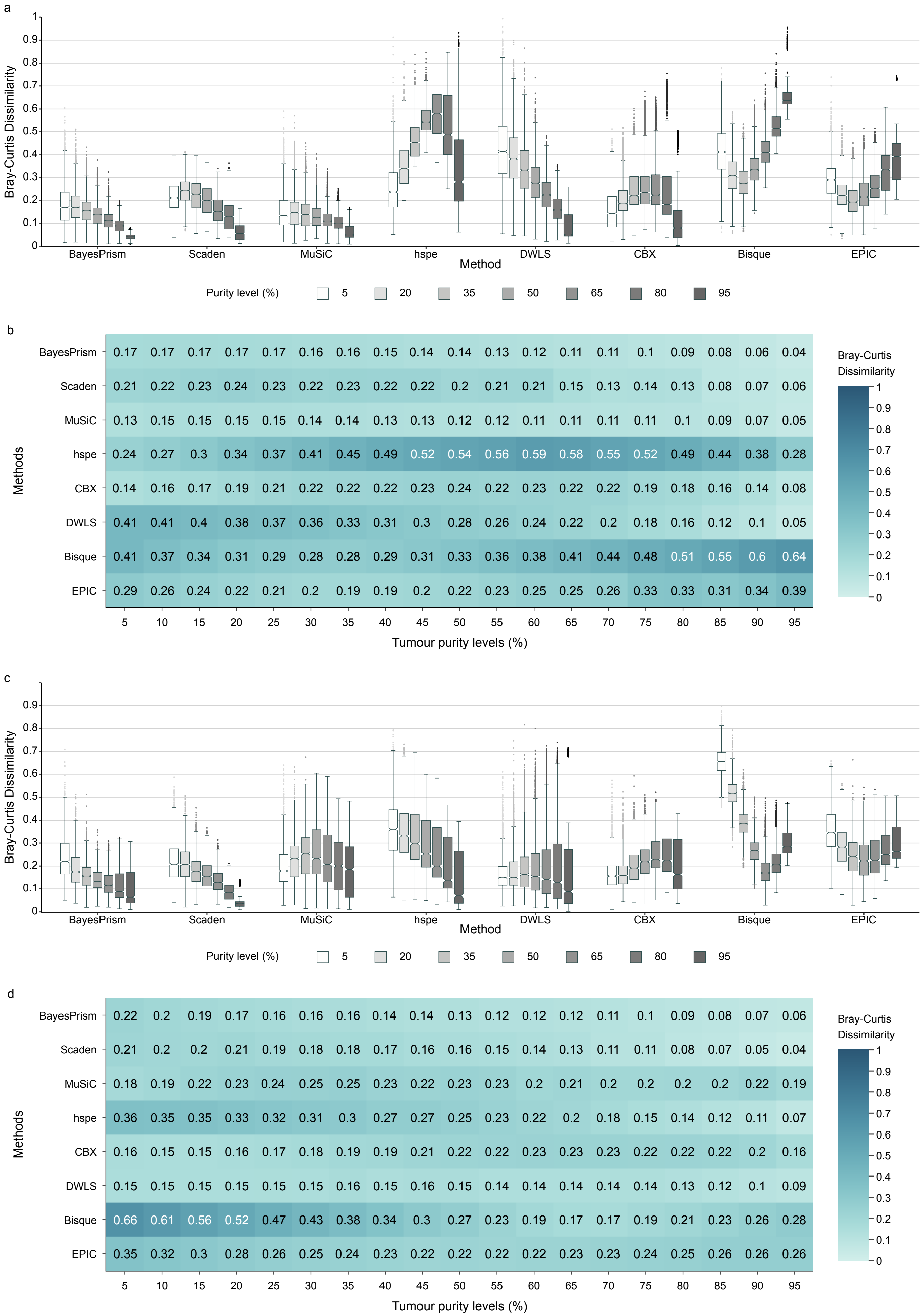
a
Bray-Curtis Dissimilarity
0 0.1 0.2 0.3 0.4 0.5 0.6 0.7 0.8 0.9 1
BayesPrism Scaden MuSiC hspe DWLS CBX Bisque EPIC
Method
Purity level (%) 5 20 35 50 65 80 95
b
Methods
Bray-Curtis Dissimilarity
BayesPrism 0.17 0.17 0.17 0.17 0.17 0.16 0.16 0.15 0.14 0.14 0.13 0.12 0.11 0.11 0.1 0.09 0.08 0.06 0.04
Scaden 0.21 0.22 0.23 0.24 0.23 0.22 0.23 0.22 0.22 0.2 0.21 0.21 0.15 0.13 0.14 0.13 0.08 0.07 0.06
MuSiC 0.13 0.15 0.15 0.15 0.15 0.14 0.14 0.13 0.13 0.12 0.12 0.11 0.11 0.11 0.11 0.1 0.09 0.07 0.05
hspe 0.24 0.27 0.3 0.34 0.37 0.41 0.45 0.49 0.52 0.54 0.56 0.59 0.58 0.55 0.52 0.49 0.44 0.38 0.28
CBX 0.14 0.16 0.17 0.19 0.21 0.22 0.22 0.22 0.23 0.24 0.22 0.23 0.22 0.22 0.19 0.18 0.16 0.14 0.08
DWLS 0.41 0.41 0.4 0.38 0.37 0.36 0.33 0.31 0.3 0.28 0.26 0.24 0.22 0.2 0.18 0.16 0.12 0.1 0.05
Bisque 0.41 0.37 0.34 0.31 0.29 0.28 0.28 0.29 0.31 0.33 0.36 0.38 0.41 0.44 0.48 0.51 0.55 0.6 0.64
EPIC 0.29 0.26 0.24 0.22 0.21 0.2 0.19 0.19 0.2 0.22 0.23 0.25 0.25 0.26 0.33 0.33 0.31 0.34 0.39
5 10 15 20 25 30 35 40 45 50 55 60 65 70 75 80 85 90 95
Tumour purity levels (%)
1 0.9 0.8 0.7 0.6 0.5 0.4 0.3 0.2 0.1 0
c
Bray-Curtis Dissimilarity
0 0.1 0.2 0.3 0.4 0.5 0.6 0.7 0.8 0.9
BayesPrism Scaden MuSiC hspe DWLS CBX Bisque EPIC
Method
Purity level (%) 5 20 35 50 65 80 95
d
Methods
Bray-Curtis Dissimilarity
BayesPrism 0.22 0.2 0.19 0.17 0.16 0.16 0.16 0.14 0.14 0.13 0.12 0.12 0.12 0.11 0.1 0.09 0.08 0.07 0.06
Scaden 0.21 0.2 0.2 0.21 0.19 0.18 0.18 0.17 0.16 0.16 0.15 0.14 0.13 0.11 0.11 0.08 0.07 0.05 0.04
MuSiC 0.18 0.19 0.22 0.23 0.24 0.25 0.25 0.23 0.22 0.23 0.23 0.2 0.21 0.2 0.2 0.2 0.2 0.22 0.19
hspe 0.36 0.35 0.35 0.33 0.32 0.31 0.3 0.27 0.27 0.25 0.23 0.22 0.2 0.18 0.15 0.14 0.12 0.11 0.07
CBX 0.16 0.15 0.15 0.16 0.17 0.18 0.19 0.19 0.21 0.22 0.22 0.23 0.23 0.23 0.22 0.22 0.22 0.2 0.16
DWLS 0.15 0.15 0.15 0.15 0.15 0.15 0.16 0.15 0.16 0.15 0.14 0.14 0.14 0.14 0.14 0.13 0.12 0.1 0.09
Bisque 0.66 0.61 0.56 0.52 0.47 0.43 0.38 0.34 0.3 0.27 0.23 0.19 0.17 0.17 0.19 0.21 0.23 0.26 0.28
EPIC 0.35 0.32 0.3 0.28 0.26 0.25 0.24 0.23 0.22 0.22 0.22 0.22 0.23 0.23 0.24 0.25 0.26 0.26 0.26
5 10 15 20 25 30 35 40 45 50 55 60 65 70 75 80 85 90 95
Tumour purity levels (%)
1 0.9 0.8 0.7 0.6 0.5 0.4 0.3 0.2 0.1 0

Supplementary Fig. 7: Overall generalisation of impact of tumour purity on deconvolutionto simulated bulk mixtures generated using scRNA-Seq from Bassez et al and Pal et al. **a)** Bray Curtis dissimilarity values predicted and ground truth cell compositions across 7 tumours purity levels (from 5% to 95%, 15% interval) using scRNA-Seq from Bassez et al. n=2,000 artificial bulk at each purity level. Each box represents the middle 50% of Bray-Curtis values, which includes the first quartile (Q1), the median, and the third quartile (Q3). Upper and lower whiskers depict maxima and minima of Bray-Curtis values, excluding outliers. Outliers are Bray-Curtis values that are more than 1.5x the interquartile range from either Q1 or Q3. Higher Bray-Curtis dissimilarity indicates poorer performance. **b)** Median Bray-Curtis dissimilarity between predicted and actual cell compositions across 19 tumours purity levels (from 5% to 95%, at 5% intervals) which are shown as proportions (x-axis) for nine computational methods (y-axis). There were 2,000 artificial bulk mixtures from Bassez et al per purity level. Darker shade of teal represents higher Bray-Curtis dissimilarity (worse performance). The numeric values are shown. Upper and lower whiskers depict Bray-Curtis values outside of the centre 50%. **c)** Bray Curtis dissimilarity predicted and ground truth cell compositions across 7 tumours purity levels (from 5% to 95%, 15% interval) using scRNA-Seq from Pal et al. n=2,000 artificial bulk at each purity level. Each box represents the middle 50% of Bray-Curtis values, which includes the first quartile (Q1), the median, and the third quartile (Q3). Upper and lower whiskers depict maxima and minima of Bray-Curtis values, excluding outliers. Outliers are Bray-Curtis values that are more than 1.5x the interquartile range from either Q1 or Q3. Higher Bray-Curtis dissimilarity indicates poorer performance. **d)** Median Bray-Curtis dissimilarity between predicted and actual cell compositions across 19 tumours purity levels (samples with tumour cells ranging from 5% to 95%, at 5% intervals) which are shown as proportions (x-axis) for nine computational methods (y-axis). There were 2,000 artificial bulk mixtures from Pal et al per purity level. Darker shade of teal represents higher Bray-Curtis dissimilarity (worse performance). The numeric values are shown. Upper and lower whiskers depict Bray-Curtis values outside of the centre 50%. For all sub-figures, deconvolution methods, excluding CPM, are organised in the same order as Fig. 2a and Supplementary Fig. 4 for comparison. Source data are provided as a Source Data file.

a

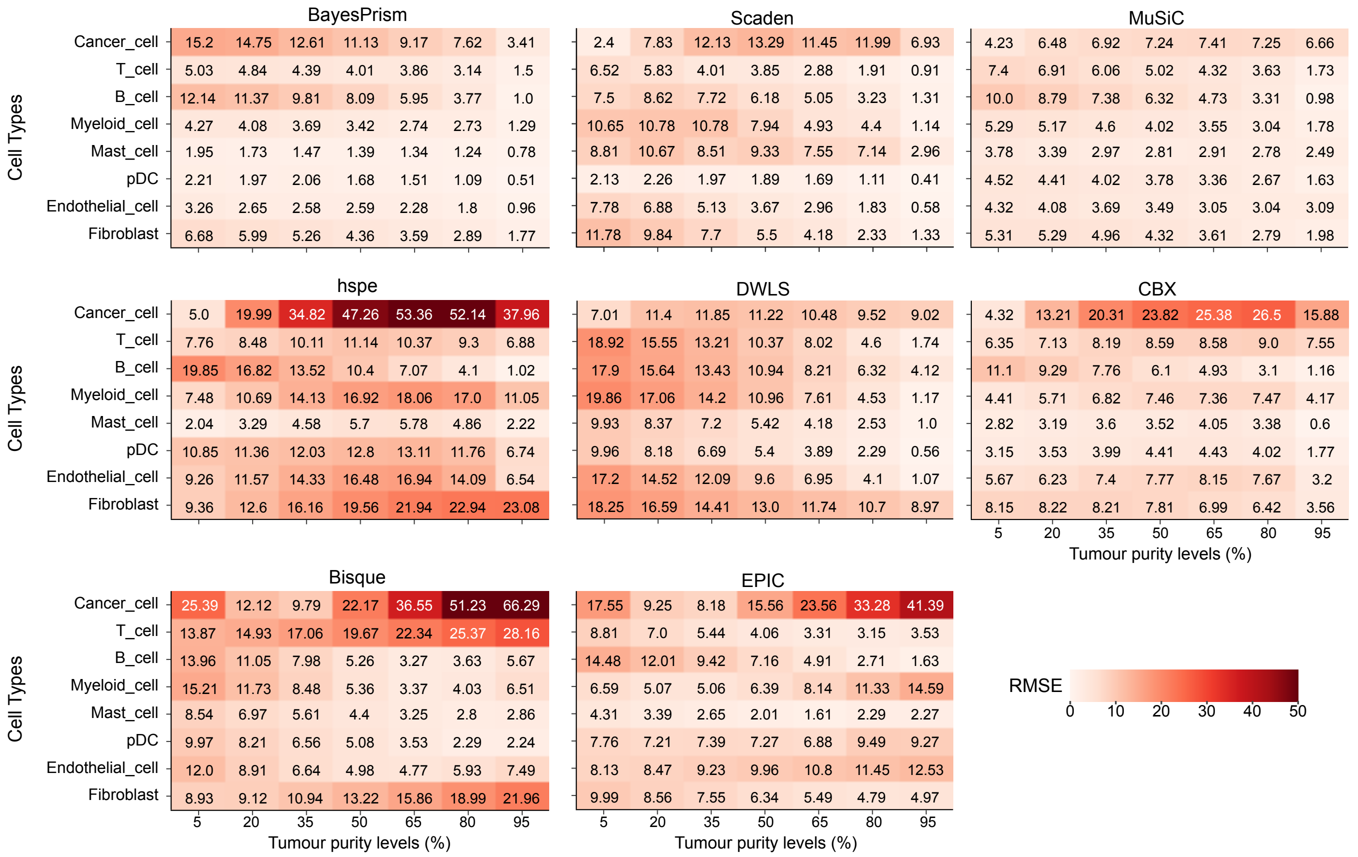
BayesPrism
Cancer_cell 15.2 14.75 12.61 11.13 9.17 7.62 3.41
T_cell 5.03 4.84 4.39 4.01 3.86 3.14 1.5
B_cell 12.14 11.37 9.81 8.09 5.95 3.77 1.0
Myeloid_cell 4.27 4.08 3.69 3.42 2.74 2.73 1.29
Mast_cell 1.95 1.73 1.47 1.39 1.34 1.24 0.78
pDC 2.21 1.97 2.06 1.68 1.51 1.09 0.51
Endothelial_cell 3.26 2.65 2.58 2.59 2.28 1.8 0.96
Fibroblast 6.68 5.99 5.26 4.36 3.59 2.89 1.77
Scaden
Cancer_cell 2.4 7.83 12.13 13.29 11.45 11.99 6.93
T_cell 6.52 5.83 4.01 3.85 2.88 1.91 0.91
B_cell 7.5 8.62 7.72 6.18 5.05 3.23 1.31
Myeloid_cell 10.65 10.78 10.78 7.94 4.93 4.4 1.14
Mast_cell 8.81 10.67 8.51 9.33 7.55 7.14 2.96
pDC 2.13 2.26 1.97 1.89 1.69 1.11 0.41
Endothelial_cell 7.78 6.88 5.13 3.67 2.96 1.83 0.58
Fibroblast 11.78 9.84 7.7 5.5 4.18 2.33 1.33
MuSiC
Cancer_cell 4.23 6.48 6.92 7.24 7.41 7.25 6.66
T_cell 7.4 6.91 6.06 5.02 4.32 3.63 1.73
B_cell 10.0 8.79 7.38 6.32 4.73 3.31 0.98
Myeloid_cell 5.29 5.17 4.6 4.02 3.55 3.04 1.78
Mast_cell 3.78 3.39 2.97 2.81 2.91 2.78 2.49
pDC 4.52 4.41 4.02 3.78 3.36 2.67 1.63
Endothelial_cell 4.32 4.08 3.69 3.49 3.05 3.04 3.09
Fibroblast 5.31 5.29 4.96 4.32 3.61 2.79 1.98
hspe
Cancer_cell 5.0 19.99 34.82 47.26 53.36 52.14 37.96
T_cell 7.76 8.48 10.11 11.14 10.37 9.3 6.88
B_cell 19.85 16.82 13.52 10.4 7.07 4.1 1.02
Myeloid_cell 7.48 10.69 14.13 16.92 18.06 17.0 11.05
Mast_cell 2.04 3.29 4.58 5.7 5.78 4.86 2.22
pDC 10.85 11.36 12.03 12.8 13.11 11.76 6.74
Endothelial_cell 9.26 11.57 14.33 16.48 16.94 14.09 6.54
Fibroblast 9.36 12.6 16.16 19.56 21.94 22.94 23.08
DWLS
Cancer_cell 7.01 11.4 11.85 11.22 10.48 9.52 9.02
T_cell 18.92 15.55 13.21 10.37 8.02 4.6 1.74
B_cell 17.9 15.64 13.43 10.94 8.21 6.32 4.12
Myeloid_cell 19.86 17.06 14.2 10.96 7.61 4.53 1.17
Mast_cell 9.93 8.37 7.2 5.42 4.18 2.53 1.0
pDC 9.96 8.18 6.69 5.4 3.89 2.29 0.56
Endothelial_cell 17.2 14.52 12.09 9.6 6.95 4.1 1.07
Fibroblast 18.25 16.59 14.41 13.0 11.74 10.7 8.97
CBX
Cancer_cell 4.32 13.21 20.31 23.82 25.38 26.5 15.88
T_cell 6.35 7.13 8.19 8.59 8.58 9.0 7.55
B_cell 11.1 9.29 7.76 6.1 4.93 3.1 1.16
Myeloid_cell 4.41 5.71 6.82 7.46 7.36 7.47 4.17
Mast_cell 2.82 3.19 3.6 3.52 4.05 3.38 0.6
pDC 3.15 3.53 3.99 4.41 4.43 4.02 1.77
Endothelial_cell 5.67 6.23 7.4 7.77 8.15 7.67 3.2
Fibroblast 8.15 8.22 8.21 7.81 6.99 6.42 3.56
Bisque
Cancer_cell 25.39 12.12 9.79 22.17 36.55 51.23 66.29
T_cell 13.87 14.93 17.06 19.67 22.34 25.37 28.16
B_cell 13.96 11.05 7.98 5.26 3.27 3.63 5.67
Myeloid_cell 15.21 11.73 8.48 5.36 3.37 4.03 6.51
Mast_cell 8.54 6.97 5.61 4.4 3.25 2.8 2.86
pDC 9.97 8.21 6.56 5.08 3.53 2.29 2.24
Endothelial_cell 12.0 8.91 6.64 4.98 4.77 5.93 7.49
Fibroblast 8.93 9.12 10.94 13.22 15.86 18.99 21.96
EPIC
Cancer_cell 17.55 9.25 8.18 15.56 23.56 33.28 41.39
T_cell 8.81 7.0 5.44 4.06 3.31 3.15 3.53
B_cell 14.48 12.01 9.42 7.16 4.91 2.71 1.63
Myeloid_cell 6.59 5.07 5.06 6.39 8.14 11.33 14.59
Mast_cell 4.31 3.39 2.65 2.01 1.61 2.29 2.27
pDC 7.76 7.21 7.39 7.27 6.88 9.49 9.27
Endothelial_cell 8.13 8.47 9.23 9.96 10.8 11.45 12.53
Fibroblast 9.99 8.56 7.55 6.34 5.49 4.79 4.97
Cell Types
5 20 35 50 65 80 95
Tumour purity levels (%)
RMSE
0 10 20 30 40 50

b

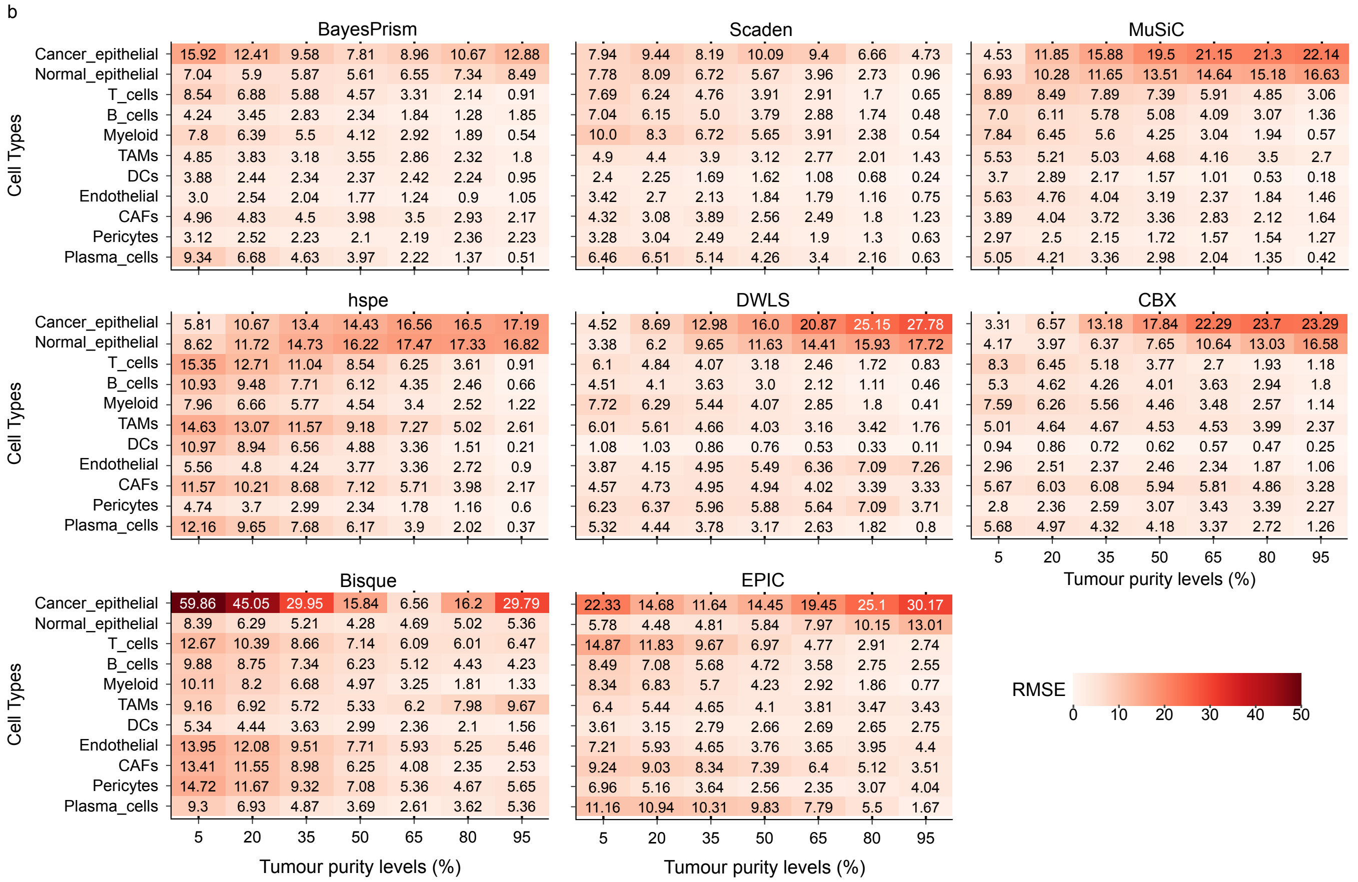
BayesPrism
Cancer_epithelial 15.92 12.41 9.58 7.81 8.96 10.67 12.88
Normal_epithelial 7.04 5.9 5.87 5.61 6.55 7.34 8.49
T_cells 8.54 6.88 5.88 4.57 3.31 2.14 0.91
B_cells 4.24 3.45 2.83 2.34 1.84 1.28 1.85
Myeloid 7.8 6.39 5.5 4.12 2.92 1.89 0.54
TAMs 4.85 3.83 3.18 3.55 2.86 2.32 1.8
DCs 3.88 2.44 2.34 2.37 2.42 2.24 0.95
Endothelial 3.0 2.54 2.04 1.77 1.24 0.9 1.05
CAFs 4.96 4.83 4.5 3.98 3.5 2.93 2.17
Pericytes 3.12 2.52 2.23 2.1 2.19 2.36 2.23
Plasma_cells 9.34 6.68 4.63 3.97 2.22 1.37 0.51
Scaden
Cancer_epithelial 7.94 9.44 8.19 10.09 9.4 6.66 4.73
Normal_epithelial 7.78 8.09 6.72 5.67 3.96 2.73 0.96
T_cells 7.69 6.24 4.76 3.91 2.91 1.7 0.65
B_cells 7.04 6.15 5.0 3.79 2.88 1.74 0.48
Myeloid 10.0 8.3 6.72 5.65 3.91 2.38 0.54
TAMs 4.9 4.4 3.9 3.12 2.77 2.01 1.43
DCs 2.4 2.25 1.69 1.62 1.08 0.68 0.24
Endothelial 3.42 2.7 2.13 1.84 1.79 1.16 0.75
CAFs 4.32 3.08 3.89 2.56 2.49 1.8 1.23
Pericytes 3.28 3.04 2.49 2.44 1.9 1.3 0.63
Plasma_cells 6.46 6.51 5.14 4.26 3.4 2.16 0.63
MuSiC
Cancer_epithelial 4.53 11.85 15.88 19.5 21.15 21.3 22.14
Normal_epithelial 6.93 10.28 11.65 13.51 14.64 15.18 16.63
T_cells 8.89 8.49 7.89 7.39 5.91 4.85 3.06
B_cells 7.0 6.11 5.78 5.08 4.09 3.07 1.36
Myeloid 7.84 6.45 5.6 4.25 3.04 1.94 0.57
TAMs 5.53 5.21 5.03 4.68 4.16 3.5 2.7
DCs 3.7 2.89 2.17 1.57 1.01 0.53 0.18
Endothelial 5.63 4.76 4.04 3.19 2.37 1.84 1.46
CAFs 3.89 4.04 3.72 3.36 2.83 2.12 1.64
Pericytes 2.97 2.5 2.15 1.72 1.57 1.54 1.27
Plasma_cells 5.05 4.21 3.36 2.98 2.04 1.35 0.42
hspe
Cancer_epithelial 5.81 10.67 13.4 14.43 16.56 16.5 17.19
Normal_epithelial 8.62 11.72 14.73 16.22 17.47 17.33 16.82
T_cells 15.35 12.71 11.04 8.54 6.25 3.61 0.91
B_cells 10.93 9.48 7.71 6.12 4.35 2.46 0.66
Myeloid 7.96 6.66 5.77 4.54 3.4 2.52 1.22
TAMs 14.63 13.07 11.57 9.18 7.27 5.02 2.61
DCs 10.97 8.94 6.56 4.88 3.36 1.51 0.21
Endothelial 5.56 4.8 4.24 3.77 3.36 2.72 0.9
CAFs 11.57 10.21 8.68 7.12 5.71 3.98 2.17
Pericytes 4.74 3.7 2.99 2.34 1.78 1.16 0.6
Plasma_cells 12.16 9.65 7.68 6.17 3.9 2.02 0.37
DWLS
Cancer_epithelial 4.52 8.69 12.98 16.0 20.87 25.15 27.78
Normal_epithelial 3.38 6.2 9.65 11.63 14.41 15.93 17.72
T_cells 6.1 4.84 4.07 3.18 2.46 1.72 0.83
B_cells 4.51 4.1 3.63 3.0 2.12 1.11 0.46
Myeloid 7.72 6.29 5.44 4.07 2.85 1.8 0.41
TAMs 6.01 5.61 4.66 4.03 3.16 3.42 1.76
DCs 1.08 1.03 0.86 0.76 0.53 0.33 0.11
Endothelial 3.87 4.15 4.95 5.49 6.36 7.09 7.26
CAFs 4.57 4.73 4.95 4.94 4.02 3.39 3.33
Pericytes 6.23 6.37 5.96 5.88 5.64 7.09 3.71
Plasma_cells 5.32 4.44 3.78 3.17 2.63 1.82 0.8
CBX
Cancer_epithelial 3.31 6.57 13.18 17.84 22.29 23.7 23.29
Normal_epithelial 4.17 3.97 6.37 7.65 10.64 13.03 16.58
T_cells 8.3 6.45 5.18 3.77 2.7 1.93 1.18
B_cells 5.3 4.62 4.26 4.01 3.63 2.94 1.8
Myeloid 7.59 6.26 5.56 4.46 3.48 2.57 1.14
TAMs 5.01 4.64 4.67 4.53 4.53 3.99 2.37
DCs 0.94 0.86 0.72 0.62 0.57 0.47 0.25
Endothelial 2.96 2.51 2.37 2.46 2.34 1.87 1.06
CAFs 5.67 6.03 6.08 5.94 5.81 4.86 3.28
Pericytes 2.8 2.36 2.59 3.07 3.43 3.39 2.27
Plasma_cells 5.68 4.97 4.32 4.18 3.37 2.72 1.26
Bisque
Cancer_epithelial 59.86 45.05 29.95 15.84 6.56 16.2 29.79
Normal_epithelial 8.39 6.29 5.21 4.28 4.69 5.02 5.36
T_cells 12.67 10.39 8.66 7.14 6.09 6.01 6.47
B_cells 9.88 8.75 7.34 6.23 5.12 4.43 4.23
Myeloid 10.11 8.2 6.68 4.97 3.25 1.81 1.33
TAMs 9.16 6.92 5.72 5.33 6.2 7.98 9.67
DCs 5.34 4.44 3.63 2.99 2.36 2.1 1.56
Endothelial 13.95 12.08 9.51 7.71 5.93 5.25 5.46
CAFs 13.41 11.55 8.98 6.25 4.08 2.35 2.53
Pericytes 14.72 11.67 9.32 7.08 5.36 4.67 5.65
Plasma_cells 9.3 6.93 4.87 3.69 2.61 3.62 5.36
EPIC
Cancer_epithelial 22.33 14.68 11.64 14.45 19.45 25.1 30.17
Normal_epithelial 5.78 4.48 4.81 5.84 7.97 10.15 13.01
T_cells 14.87 11.83 9.67 6.97 4.77 2.91 2.74
B_cells 8.49 7.08 5.68 4.72 3.58 2.75 2.55
Myeloid 8.34 6.83 5.7 4.23 2.92 1.86 0.77
TAMs 6.4 5.44 4.65 4.1 3.81 3.47 3.43
DCs 3.61 3.15 2.79 2.66 2.69 2.65 2.75
Endothelial 7.21 5.93 4.65 3.76 3.65 3.95 4.4
CAFs 9.24 9.03 8.34 7.39 6.4 5.12 3.51
Pericytes 6.96 5.16 3.64 2.56 2.35 3.07 4.04
Plasma_cells 11.16 10.94 10.31 9.83 7.79 5.5 1.67
Cell Types
5 20 35 50 65 80 95
Tumour purity levels (%)
RMSE
0 10 20 30 40 50

Supplementary Fig. 8: Cell-type-specific generalisation of impact of tumour purity on deconvolution to simulated bulk mixtures generated using scRNA-Seq from Bassez et al and Pal et al. a) Median RMSE between predicted and actual cell compositions of simulated bulk mixtures generated using scRNA-Seq from Bassez et al, aggregated by cell type. Seven tumour purity levels are shown (from 5% to 95%, 15% interval). Darker shade of red represents higher RMSE values (worse performance), with numeric RSME values shown. Major cell types (y-axis) are organised into three categories: cancer (Cancer_cell), immune (T_cell, B_cell, Myeloid_cell, Mast_cell and pDC), and stromal cells (Endothelial_cell and Fibroblast). **b)** RMSE between predicted and actual cell compositions of simulated bulk mixtures generated using scRNA-Seq from Bassez et al, aggregated by cell type. Seven tumour purity levels are shown (from 5% to 95%, 15% interval). Darker shade of red represents higher RMSE values (worse performance), with numeric RSME values shown. Major cell types (y-axis) are organised into three categories: epithelial (Cancer_epithelial and Normal_epithelial), immune (T_cells, B_cells, Myeloid, TAMs and DCs), and stromal cells (Endothelial, CAFs, Pericytes and Plasma_cells). TAMs: tumour-associated macrophage, DCs: dendritic cells, CAFs: cancer-associated fibroblast, pDC: plasmacytoid dendritic cell, RMSE: Root Mean Square Error, scRNA-Seq: Single-cell RNA Sequencing. Source data are provided as a Source Data file.

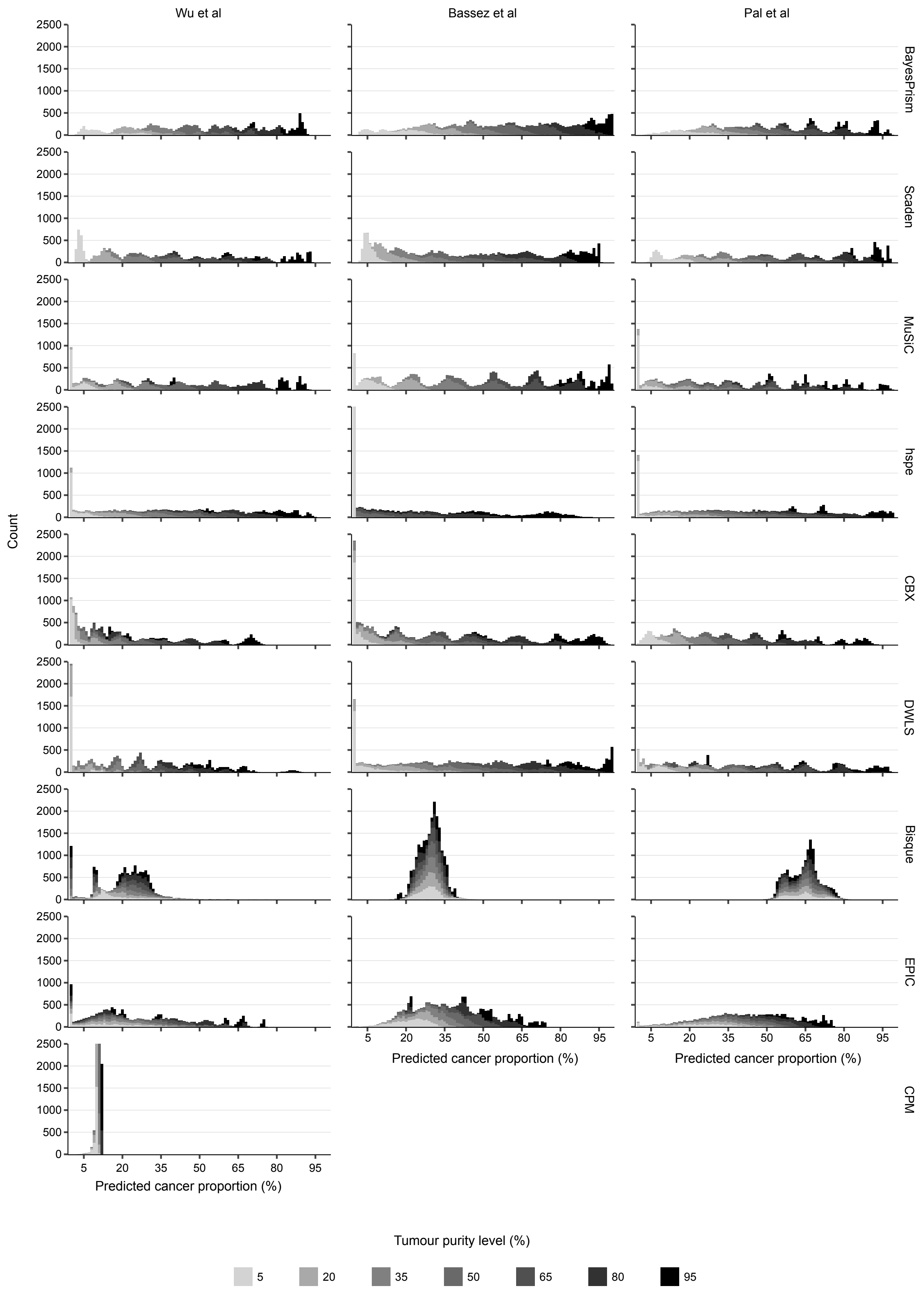

Wu et al
Bassez et al
Pal et al
BayesPrism
Scaden
MuSiC
hspe
CBX
DWLS
Bisque
EPIC
CPM
Count
0
500
1000
1500
2000
2500
5
20
35
50
65
80
95
Predicted cancer proportion (%)
Tumour purity level (%)

Supplementary Fig. 9: Histogram of predicted cancer proportions. Histogram distributions (*bin_size*=100) of predicted cancer proportions from simulated mixtures generated using single cells from Wu et al (left column), Bassez et al (middle column) and Pal et al (right column). Each histogram is aggregated and coloured by 7 tumour purity levels (from 5% to 95%, 15% interval, depicted as tick values on x-axes), and represents performance of each method in estimating cancer populations. x-axes represent predicted cancer proportions by each method, aggregated into 100 bins between 0% and 100% (i.e. bin width is 1%). y-axes represent count of the predicted bin on x-axis. The more accurate the prediction, the more each coloured histogram will be centred at the corresponding tumour purity level. A perfectly predicting model would produce 7 straight lines on top of the 7 annotated tumour purity levels. Source data are provided as a Source Data file.

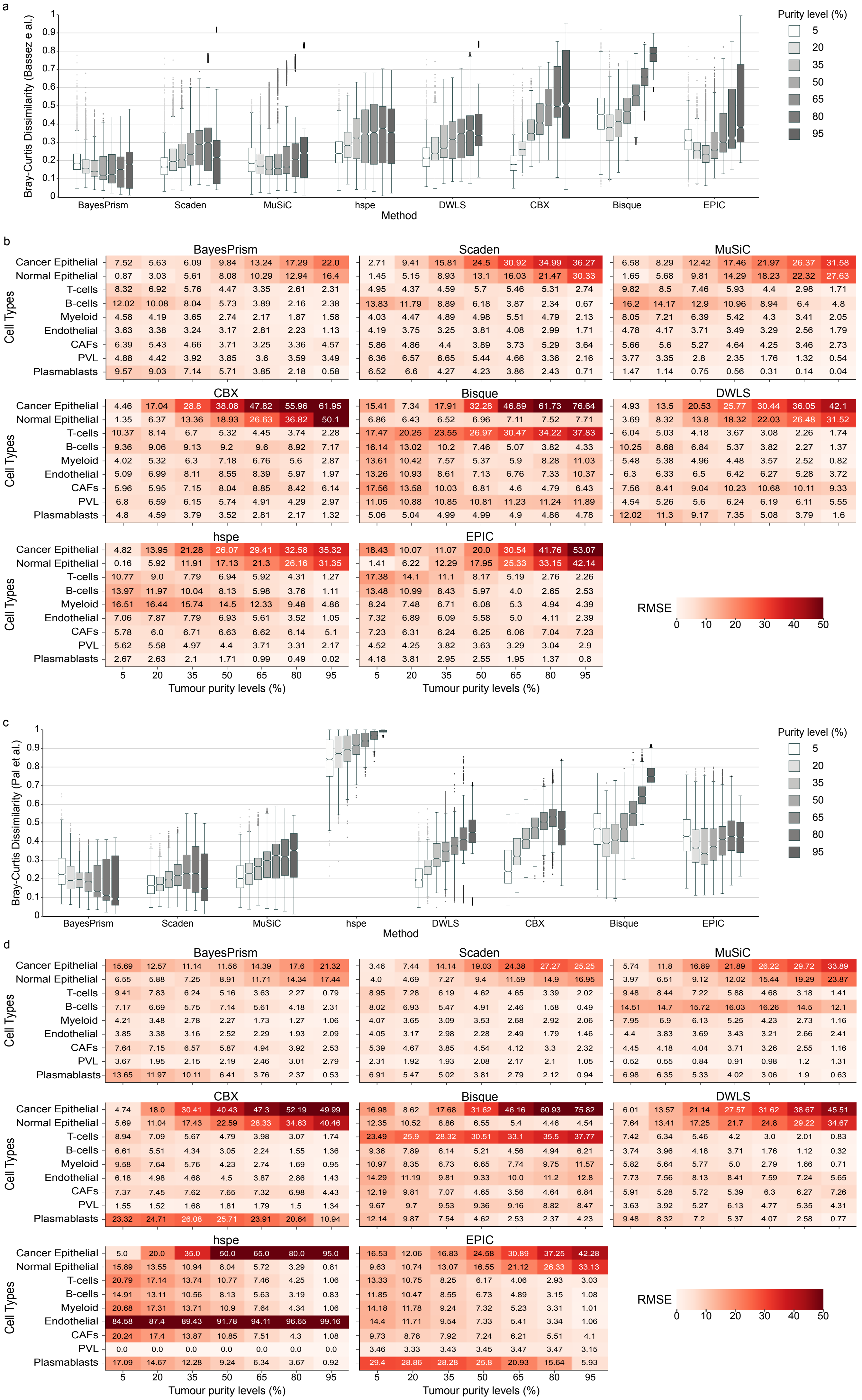

a
Bray-Curtis Dissimilarity (Bassez e al.)
1
0.9
0.8
0.7
0.6
0.5
0.4
0.3
0.2
0.1
0
BayesPrism
Scaden
MuSiC
hspe
DWLS
CBX
Bisque
EPIC
Method
Purity level (%)
5
20
35
50
65
80
95
b
BayesPrism
Cancer Epithelial 7.52 5.63 6.09 9.84 13.24 17.29 22.0
Normal Epithelial 0.87 3.03 5.61 8.08 10.29 12.94 16.4
T-cells 8.32 6.92 5.76 4.47 3.35 2.61 2.31
B-cells 12.02 10.08 8.04 5.73 3.89 2.16 2.38
Myeloid 4.58 4.19 3.65 2.74 2.17 1.87 1.58
Endothelial 3.63 3.38 3.24 3.17 2.81 2.23 1.13
CAFs 6.39 5.43 4.66 3.71 3.25 3.36 4.57
PVL 4.88 4.42 3.92 3.85 3.6 3.59 3.49
Plasmablasts 9.57 9.03 7.14 5.71 3.85 2.18 0.58
Scaden
2.71 9.41 15.81 24.5 30.92 34.99 36.27
1.45 5.15 8.93 13.1 16.03 21.47 30.33
4.95 4.37 4.59 5.7 5.46 5.31 2.74
13.83 11.79 8.89 6.18 3.87 2.34 0.67
4.03 4.47 4.89 4.98 5.51 4.79 2.13
4.19 3.75 3.25 3.81 4.08 2.99 1.71
5.86 4.86 4.4 3.89 3.73 5.29 3.64
6.36 6.57 6.65 5.44 4.66 3.36 2.16
6.52 6.6 4.27 4.23 3.86 2.43 0.71
MuSiC
6.58 8.29 12.42 17.46 21.97 26.37 31.58
1.65 5.68 9.81 14.29 18.23 22.32 27.63
9.82 8.5 7.46 5.93 4.4 2.98 1.71
16.2 14.17 12.9 10.96 8.94 6.4 4.8
8.05 7.21 6.39 5.42 4.3 3.41 2.05
4.78 4.17 3.71 3.49 3.29 2.56 1.79
5.66 5.6 5.27 4.64 4.25 3.46 2.73
3.77 3.35 2.8 2.35 1.76 1.32 0.54
1.47 1.14 0.75 0.56 0.31 0.14 0.04
Cell Types
CBX
Cancer Epithelial 4.46 17.04 28.8 38.08 47.82 55.96 61.95
Normal Epithelial 1.35 6.37 13.36 18.93 26.63 36.82 50.1
T-cells 10.37 8.14 6.7 5.32 4.45 3.74 2.28
B-cells 9.36 9.06 9.13 9.2 9.6 8.92 7.17
Myeloid 4.02 5.32 6.3 7.18 6.76 5.6 2.87
Endothelial 5.09 6.99 8.11 8.55 8.39 5.97 1.97
CAFs 5.96 5.95 7.15 8.04 8.85 8.42 6.14
PVL 6.8 6.59 6.15 5.74 4.91 4.29 2.97
Plasmablasts 4.8 4.59 3.79 3.52 2.81 2.17 1.32
Bisque
15.41 7.34 17.91 32.28 46.89 61.73 76.64
6.86 6.43 6.52 6.96 7.11 7.52 7.71
17.47 20.25 23.55 26.97 30.47 34.22 37.83
16.14 13.02 10.2 7.46 5.07 3.82 4.33
13.61 10.42 7.57 5.37 5.9 8.28 11.03
13.26 10.93 8.61 7.13 6.76 7.33 10.37
17.56 13.58 10.03 6.81 4.6 4.79 6.43
11.05 10.88 10.85 10.81 11.23 11.24 11.89
5.06 5.04 4.99 4.99 4.9 4.86 4.78
DWLS
4.93 13.5 20.53 25.77 30.44 36.05 42.1
3.69 8.32 13.8 18.32 22.03 26.48 31.52
6.04 5.03 4.18 3.67 3.08 2.26 1.74
10.25 8.68 6.84 5.37 3.82 2.27 1.37
5.48 5.38 4.96 4.48 3.57 2.52 0.82
6.3 6.33 6.5 6.42 6.27 5.28 3.72
7.56 8.41 9.04 10.23 10.68 10.11 9.33
4.54 5.26 5.6 6.24 6.19 6.11 5.55
12.02 11.3 9.17 7.35 5.08 3.79 1.6
Cell Types
hspe
Cancer Epithelial 4.82 13.95 21.28 26.07 29.41 32.58 35.32
Normal Epithelial 0.16 5.92 11.91 17.13 21.3 26.16 31.35
T-cells 10.77 9.0 7.79 6.94 5.92 4.31 1.27
B-cells 13.97 11.97 10.04 8.13 5.98 3.76 1.11
Myeloid 16.51 16.44 15.74 14.5 12.33 9.48 4.86
Endothelial 7.06 7.87 7.79 6.93 5.61 3.52 1.05
CAFs 5.78 6.0 6.71 6.63 6.62 6.14 5.1
PVL 5.62 5.58 4.97 4.4 3.71 3.31 2.17
Plasmablasts 2.67 2.63 2.1 1.71 0.99 0.49 0.02
EPIC
18.43 10.07 11.07 20.0 30.54 41.76 53.07
1.41 6.22 12.29 17.95 25.33 33.15 42.14
17.38 14.1 11.1 8.17 5.19 2.76 2.26
13.48 10.99 8.43 5.97 4.0 2.65 2.53
8.24 7.48 6.71 6.08 5.3 4.94 4.39
7.32 6.89 6.09 5.58 5.0 4.11 2.39
7.23 6.31 6.24 6.25 6.06 7.04 7.23
4.52 4.25 3.82 3.63 3.29 3.04 2.9
4.18 3.81 2.95 2.55 1.95 1.37 0.8
Cell Types
5 20 35 50 65 80 95
Tumour purity levels (%)
5 20 35 50 65 80 95
Tumour purity levels (%)
RMSE
0 10 20 30 40 50
c
Bray-Curtis Dissimilarity (Pal et al.)
1
0.9
0.8
0.7
0.6
0.5
0.4
0.3
0.2
0.1
0
BayesPrism
Scaden
MuSiC
hspe
DWLS
CBX
Bisque
EPIC
Method
Purity level (%)
5
20
35
50
65
80
95
d
BayesPrism
Cancer Epithelial 15.69 12.57 11.14 11.56 14.39 17.6 21.32
Normal Epithelial 6.55 5.88 7.25 8.91 11.71 14.34 17.44
T-cells 9.41 7.83 6.24 5.16 3.63 2.27 0.79
B-cells 7.17 6.69 5.75 7.14 5.61 4.18 2.31
Myeloid 4.21 3.48 2.78 2.27 1.73 1.27 1.06
Endothelial 3.85 3.38 3.16 2.52 2.29 1.93 2.09
CAFs 7.64 7.15 6.57 5.87 4.94 3.92 2.53
PVL 3.67 1.95 2.15 2.19 2.46 3.01 2.79
Plasmablasts 13.65 11.97 10.11 6.41 3.76 2.37 0.53
Scaden
3.46 7.44 14.14 19.03 24.38 27.27 25.25
4.0 4.69 7.27 9.4 11.59 14.9 16.95
8.95 7.28 6.19 4.62 4.65 3.39 2.02
8.02 6.93 5.47 4.91 2.46 1.58 0.49
4.07 3.65 3.09 3.53 2.68 2.92 2.06
4.05 3.17 2.98 2.28 2.49 1.79 1.46
5.39 4.67 3.85 4.54 4.12 3.3 2.32
2.31 1.92 1.93 2.08 2.17 2.1 1.05
6.91 5.47 5.02 3.81 2.79 2.12 0.94
MuSiC
5.74 11.8 16.89 21.89 26.22 29.72 33.89
3.97 6.51 9.12 12.02 15.44 19.29 23.87
9.48 8.44 7.22 5.88 4.68 3.18 1.41
14.51 14.7 15.72 16.03 16.26 14.5 12.1
7.95 6.9 6.13 5.25 4.23 2.73 1.16
4.4 3.83 3.69 3.43 3.21 2.66 2.41
4.45 4.18 4.04 3.71 3.26 2.55 1.16
0.52 0.55 0.84 0.91 0.98 1.2 1.31
6.98 6.35 5.33 4.02 3.06 1.9 0.63
Cell Types
CBX
Cancer Epithelial 4.74 18.0 30.41 40.43 47.3 52.19 49.99
Normal Epithelial 5.69 11.04 17.43 22.59 28.33 34.63 40.46
T-cells 8.94 7.09 5.67 4.79 3.98 3.07 1.74
B-cells 6.61 5.51 4.34 3.05 2.24 1.55 1.36
Myeloid 9.58 7.64 5.76 4.23 2.74 1.69 0.95
Endothelial 6.18 4.98 4.68 4.5 3.87 2.86 1.43
CAFs 7.37 7.45 7.62 7.65 7.32 6.98 4.43
PVL 1.55 1.52 1.68 1.81 1.79 1.5 1.34
Plasmablasts 23.32 24.71 26.08 25.71 23.91 20.64 10.94
Bisque
16.98 8.62 17.68 31.62 46.16 60.93 75.82
12.35 10.52 8.86 6.55 5.4 4.46 4.54
23.49 25.9 28.32 30.51 33.1 35.5 37.77
9.36 7.89 6.14 5.21 4.56 4.94 6.21
10.97 8.35 6.73 6.65 7.74 9.75 11.57
14.29 11.19 9.81 9.33 10.0 11.2 12.8
12.19 9.81 7.07 4.65 3.56 4.64 6.84
9.67 9.7 9.53 9.36 9.16 8.82 8.47
12.14 9.87 7.54 4.62 2.53 2.37 4.23
DWLS
6.01 13.57 21.14 27.57 31.62 38.67 45.51
7.64 13.41 17.25 21.7 24.8 29.22 34.67
7.42 6.34 5.46 4.2 3.0 2.01 0.83
3.74 3.96 4.18 3.71 1.76 1.12 0.32
5.82 5.64 5.77 5.0 2.79 1.66 0.71
7.73 7.56 8.13 8.41 7.59 7.24 5.65
5.91 5.28 5.72 5.39 6.3 6.27 7.26
3.63 3.92 5.27 6.13 4.77 5.35 4.31
9.48 8.32 7.2 5.37 4.07 2.58 0.77
Cell Types
hspe
Cancer Epithelial 5.0 20.0 35.0 50.0 65.0 80.0 95.0
Normal Epithelial 15.89 13.55 10.94 8.04 5.72 3.29 0.81
T-cells 20.79 17.14 13.74 10.77 7.46 4.25 1.06
B-cells 14.91 13.11 10.56 8.13 5.63 3.19 0.83
Myeloid 20.68 17.31 13.71 10.9 7.64 4.34 1.06
Endothelial 84.58 87.4 89.43 91.78 94.11 96.65 99.16
CAFs 20.24 17.4 13.87 10.85 7.51 4.3 1.08
PVL 0.0 0.0 0.0 0.0 0.0 0.0 0.0
Plasmablasts 17.09 14.67 12.28 9.24 6.34 3.67 0.92
EPIC
16.53 12.06 16.83 24.58 30.89 37.25 42.28
9.63 10.74 13.07 16.55 21.12 26.33 33.13
13.33 10.75 8.25 6.17 4.06 2.93 3.03
11.85 10.47 8.55 6.73 4.89 3.15 1.08
14.18 11.78 9.24 7.32 5.23 3.31 1.01
14.4 11.71 9.54 7.33 5.41 3.34 1.06
9.73 8.78 7.92 7.24 6.21 5.51 4.1
3.46 3.33 3.43 3.45 3.47 3.47 3.15
29.4 28.86 28.28 25.8 20.93 15.64 5.93
Cell Types
5 20 35 50 65 80 95
Tumour purity levels (%)
5 20 35 50 65 80 95
Tumour purity levels (%)
RMSE
0 10 20 30 40 50

Supplementary Fig. 10: Studying the impact of tumour purity on deconvolution to simulated bulk mixtures generated using scRNA-Seq from Bassez et al and Pal et al, and single-cell reference from Wu et al. a) Bray Curtis dissimilarity predicted and ground truth cell compositions across 7 tumours purity levels (from 5% to 95%, 15% interval) using scRNA-Seq from Bassez et al. n=2,000 artificial bulk at each purity level. Each box represents the middle 50% of Bray-Curtis values, which includes the first quartile (Q1), the median, and the third quartile (Q3). Upper and lower whiskers depict maxima and minima of Bray-Curtis values, excluding outliers. Outliers are Bray-Curtis values that are more than 1.5x the interquartile range from either Q1 or Q3. Higher Bray-Curtis dissimilarity indicates poorer performance. **b)** RMSE between predicted and actual cell compositions of artificial bulk mixtures from Bassez et al, aggregated by cell type. Seven tumour purity levels are shown (from 5% to 95%, 15% interval). Darker shade of red represents higher RMSE values (worse performance), with numeric RSME values shown. Major cell types (y-axis) are organised into three categories: epithelial (Cancer_epithelial and Normal_epithelial), immune (T_cells, B_cells, Myeloid, TAMs and DCs), and stromal cells (Endothelial, CAFs, Pericytes and Plasma_cells). **c)** Bray Curtis dissimilarity predicted and ground truth cell compositions across 7 tumours purity levels (from 5% to 95%, 15% interval) using scRNA-Seq from Pal et al. Deconvolution methods, excluding CPM, are organised in the same order as Fig. 2a for comparison. n=2,000 artificial bulk at each purity level. Each box represents the middle 50% of Bray-Curtis values, which includes the first quartile (Q1), the median, and the third quartile (Q3). Upper and lower whiskers depict maxima and minima of Bray-Curtis values, excluding outliers. Outliers are Bray-Curtis values that are more than 1.5x the interquartile range from either Q1 or Q3. Higher Bray-Curtis dissimilarity indicates poorer performance. **d)** RMSE between predicted and actual cell compositions of artificial bulk mixtures from Pal et al, aggregated by cell type. Seven tumour purity levels are shown (from 5% to 95%, 15% interval). Darker shade of red represents higher RMSE values (worse performance), with numeric RSME values shown. Major cell types (y-axis) are organised into three categories: epithelial (Cancer_epithelial and Normal_epithelial), immune (T_cells, B_cells, Myeloid, TAMs and DCs), and stromal cells (Endothelial, CAFs, Pericytes and Plasma_cells). TAMs: tumour-associated macrophage, DCs: dendritic cells, CAFs: cancer-associated fibroblast, RMSE: Root Mean Square Error, scRNA-Seq: Single-cell RNA Sequencing. Source data are provided as a Source Data file.

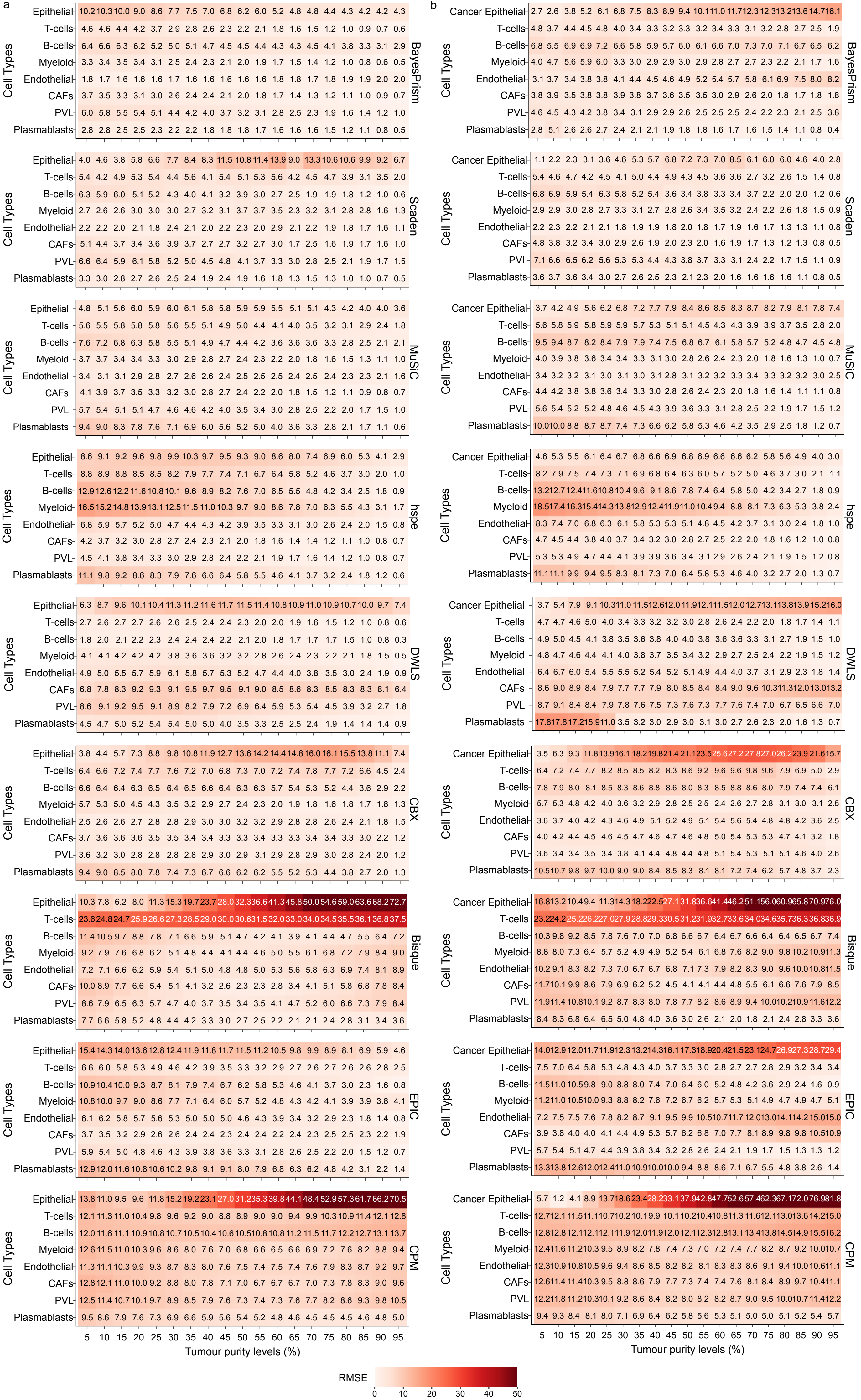
a
b
Epithelial
Cancer Epithelial
T-cells
B-cells
Myeloid
Endothelial
CAFs
PVL
Plasmablasts
Cell Types
BayesPrism
Scaden
MuSiC
hspe
DWLS
CBX
Bisque
EPIC
CPM
5 10 15 20 25 30 35 40 45 50 55 60 65 70 75 80 85 90 95
Tumour purity levels (%)
RMSE
0 10 20 30 40 50

Supplementary Fig. 11: Cell-type specific deconvolution performance for nine methods with tumour and normal epithelial cells grouped as epithelial. a) Heatmap of RMSE of eight cell types across 2,000 mixtures and 19 tumour purity levels (from 5% to 95%, 5% interval) with normal epithelial and cancer epithelial predictions collapsed into 'epithelial' cell type. **b)** Heatmap of RMSE of eight cell types across 2,000 artificial mixtures and 19 tumour purity levels (from 5% to 95%, 5% interval) with no normal epithelial cells. For both **a)** and **b)**, darker shade of red represents higher RMSE values and worse deconvolution performance. The numeric RSME values are shown. RMSE: Root Mean Square Error. Source data are provided as a Source Data file.

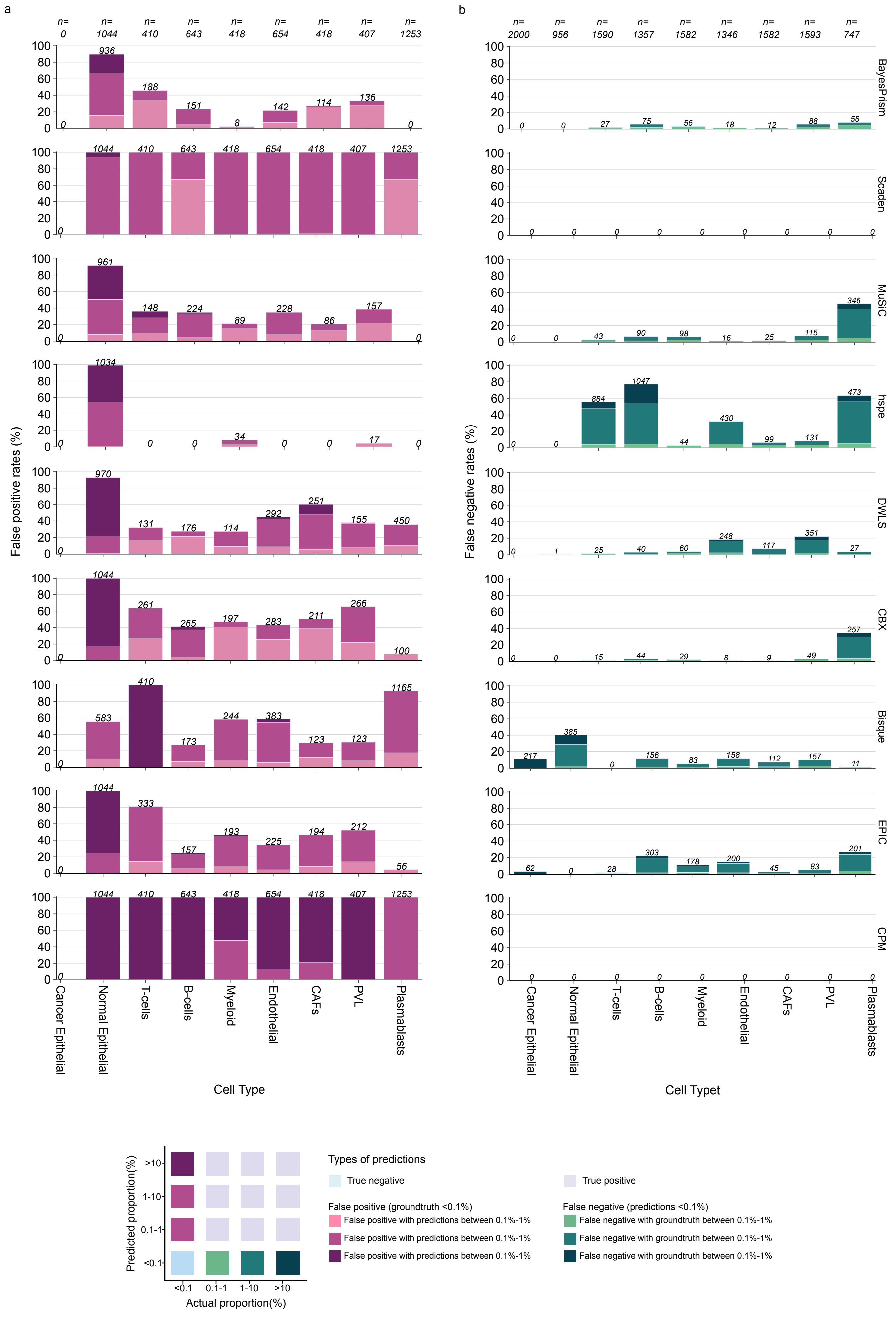
a
b
False positive rates (%)
False negative rates (%)
BayesPrism
Scaden
MuSIC
hspe
DWLS
CBX
Bisque
EPIC
CPM
Cancer Epithelial
Normal Epithelial
T-cells
B-cells
Myeloid
Endothelial
CAFs
PVL
Plasmablasts
Cell Type
Cell Typet
Predicted proportion(%)
Actual proportion(%)
Types of predictions
True negative
True positive
False positive (groundtruth <0.1%)
False positive with predictions between 0.1%-1%
False positive with predictions between 0.1%-1%
False positive with predictions between 0.1%-1%
False negative (predictions <0.1%)
False negative with groundtruth between 0.1%-1%
False negative with groundtruth between 0.1%-1%
False negative with groundtruth between 0.1%-1%

Supplementary Fig. 12: The performance of the nine deconvolution methods assessed by false positive and false negative rates. a) Percentages of the three levels of false positives out of the total number of false positives and true negatives (actual proportion less than 0.1%). Percentages and counts (shown above each bar) of false positives are shown for each individual cell type. The total number of missing cell type components used to determine false positive rates for all methods is shown at the top with the "*n*=" prefix. **b)** Percentages of the three levels of false negatives out of total number of all false negatives and true positives (predicted proportions less than 0.1%). Percentages and counts (shown above each bar) of false negatives are shown for each individual cell type. The total number of cell type components used to determine false negative rates for all methods is shown at the top with the "*n*=" prefix. Figure legend illustrates definitions of true negative, false positive, true positive, and false negative predictions. Source data are provided as a Source Data file.

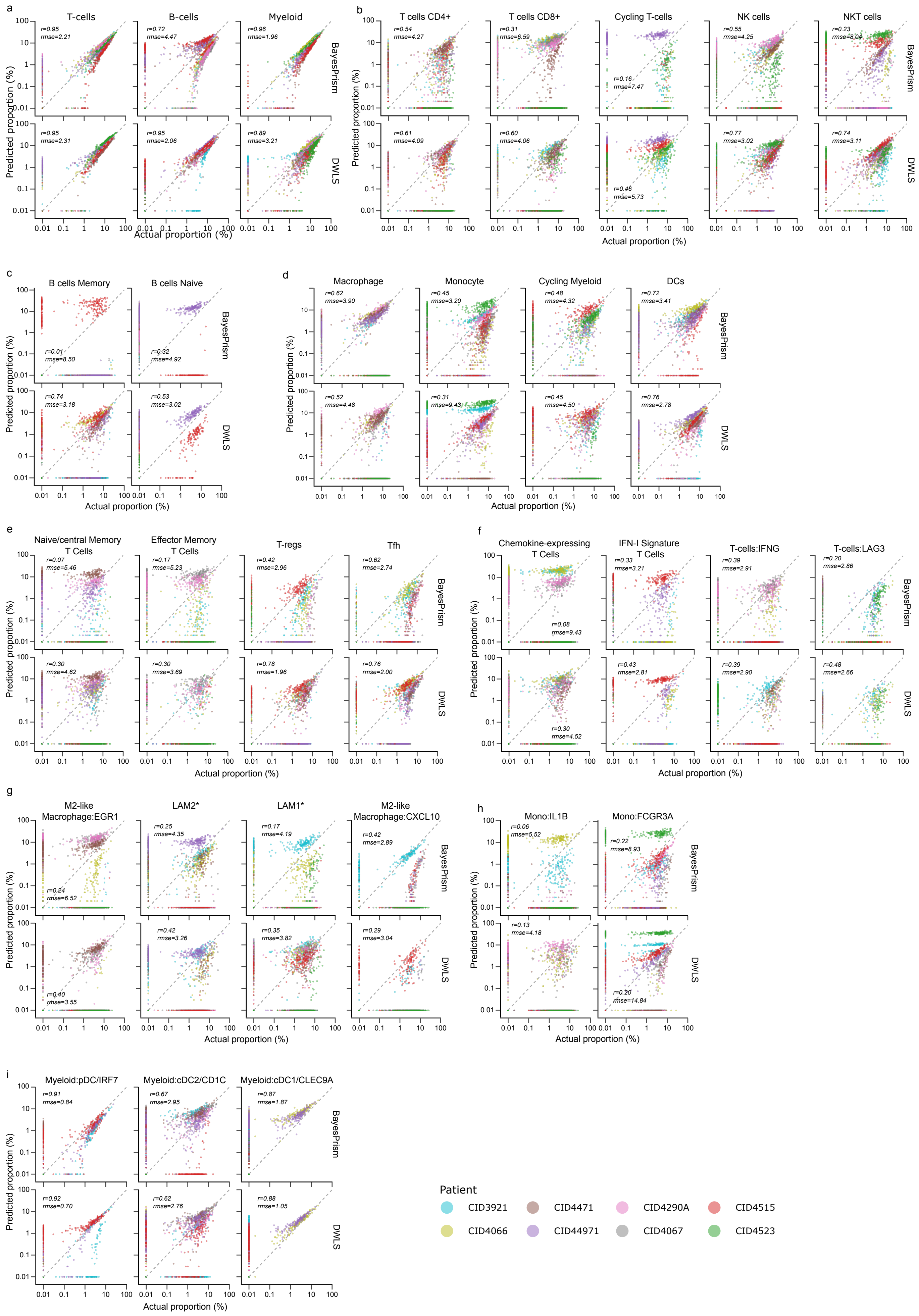
a
T-cells
B-cells
Myeloid
BayesPrism
DWLS
r=0.95 rmse=2.21
r=0.72 rmse=4.47
r=0.96 rmse=1.96
r=0.95 rmse=2.31
r=0.95 rmse=2.06
r=0.89 rmse=3.21
Predicted proportion (%)
Actual proportion (%)
b
T cells CD4+
T cells CD8+
Cycling T-cells
NK cells
NKT cells
r=0.54 rmse=4.27
r=0.31 rmse=6.59
r=0.16 rmse=7.47
r=0.55 rmse=4.25
r=0.23 rmse=8.04
r=0.61 rmse=4.09
r=0.60 rmse=4.06
r=0.46 rmse=5.73
r=0.77 rmse=3.02
r=0.74 rmse=3.11
c
B cells Memory
B cells Naive
r=0.01 rmse=8.50
r=0.32 rmse=4.92
r=0.74 rmse=3.18
r=0.53 rmse=3.02
d
Macrophage
Monocyte
Cycling Myeloid
DCs
r=0.62 rmse=3.90
r=0.45 rmse=3.20
r=0.48 rmse=4.32
r=0.72 rmse=3.41
r=0.52 rmse=4.48
r=0.31 rmse=9.43
r=0.45 rmse=4.50
r=0.76 rmse=2.78
e
Naive/central Memory T Cells
Effector Memory T Cells
T-regs
Tfh
r=0.07 rmse=5.46
r=0.17 rmse=5.23
r=0.42 rmse=2.96
r=0.62 rmse=2.74
r=0.30 rmse=4.62
r=0.30 rmse=3.69
r=0.78 rmse=1.96
r=0.76 rmse=2.00
f
Chemokine-expressing T Cells
IFN-I Signature T Cells
T-cells:IFNG
T-cells:LAG3
r=0.08 rmse=9.43
r=0.33 rmse=3.21
r=0.39 rmse=2.91
r=0.20 rmse=2.86
r=0.30 rmse=4.52
r=0.43 rmse=2.81
r=0.39 rmse=2.90
r=0.48 rmse=2.66
g
M2-like Macrophage:EGR1
LAM2*
LAM1*
M2-like Macrophage:CXCL10
r=0.24 rmse=6.52
r=0.25 rmse=4.35
r=0.17 rmse=4.19
r=0.42 rmse=2.89
r=0.40 rmse=3.55
r=0.42 rmse=3.26
r=0.35 rmse=3.82
r=0.29 rmse=3.04
h
Mono:IL1B
Mono:FCGR3A
r=0.06 rmse=5.52
r=0.22 rmse=8.93
r=0.13 rmse=4.18
r=0.20 rmse=14.84
i
Myeloid:pDC/IRF7
Myeloid:cDC2/CD1C
Myeloid:cDC1/CLEC9A
r=0.91 rmse=0.84
r=0.67 rmse=2.95
r=0.87 rmse=1.87
r=0.92 rmse=0.70
r=0.62 rmse=2.76
r=0.88 rmse=1.05
Patient
CID3921
CID4471
CID4290A
CID4515
CID4066
CID44971
CID4067
CID4523

Supplementary Fig. 13: Cross-lineage performance of BayesPrism and DWLS by patient. Scatter plots of predicted (y-axis) versus actual (x-axis) cell compositions of BayesPrism and DWLS for major **(a)**, minor **(b-d)** and subset immune cell types **(e-i)**. Each point represents a test mixture component, with its colour representing one of eight test patients (from which single cells were used to generate the mixture). Dotted 45-degree diagonal line represents perfect prediction where predicted proportions match actual proportions. Cell types represented in each plot are three major immune cell types **(a)**, five minor cell types of T-cells **(b)**, two minor cell types of B-cells **(c)**, four minor myeloid cell types **(d)**, four subset cell types of CD8+ T-cells **(e)**, four subset cell types of CD4+ T-cells **(f)**, four subset macrophage cell types **(g)**, two subset monocyte cell types **(h)**, and three subset cell types of dendritic cells **(i)**. Each scatterplot is annotated with the associated Pearson's r correlation coefficient and RMSE values. RMSE: Root Mean Square Error. Source data are provided as a Source Data file.

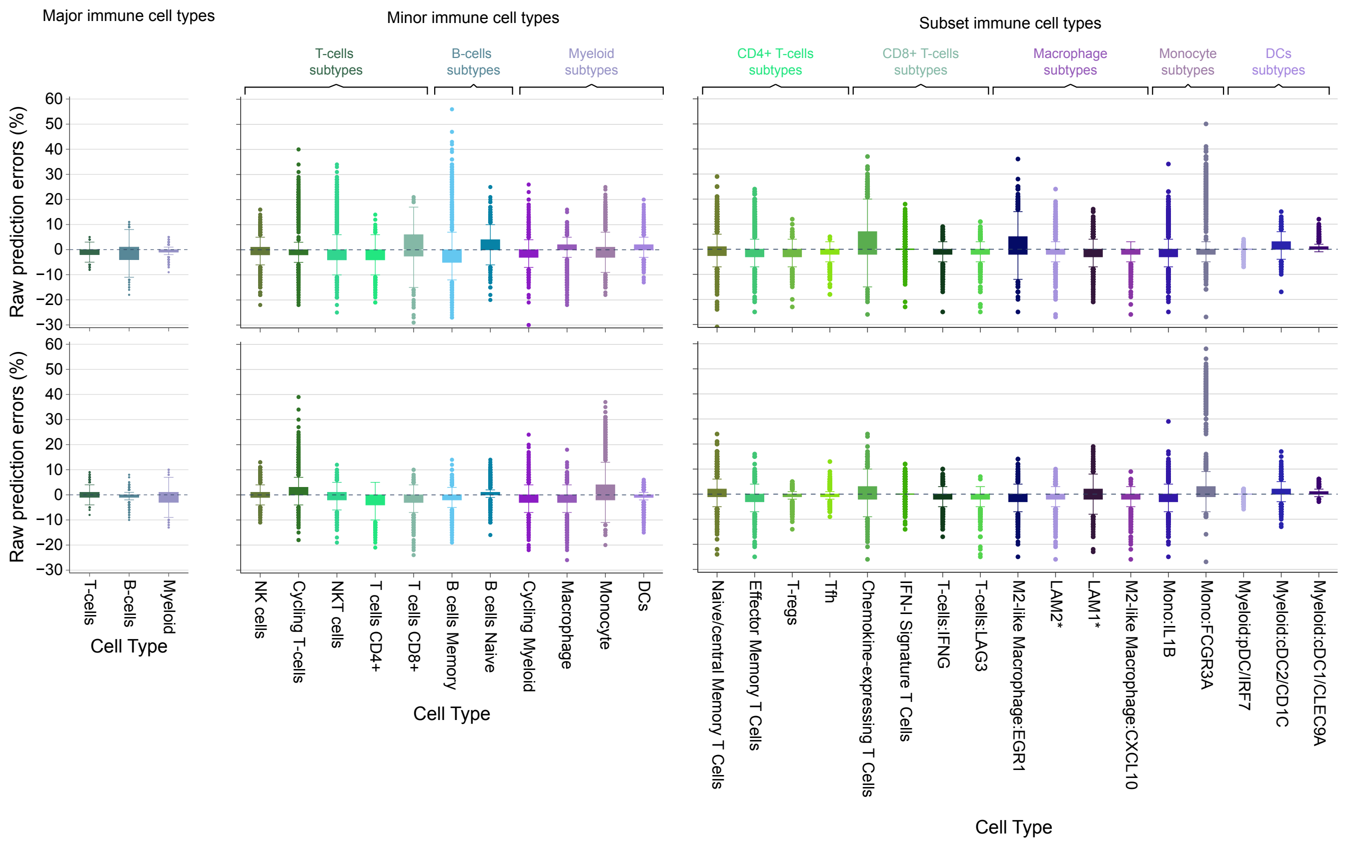

Supplementary Fig. 14: Raw prediction errors of BayesPrism and DWLS across immune lineages. Raw prediction errors (Predicted – Actual Cell Fractions) between predicted and ground truth cell compositions for BayesPrism (top) and DWLS (bottom). n=2,000 artificial bulk mixtures with 50% tumour purity. Higher positive and lower negative raw prediction errors represent poorer performance. Raw prediction error values are aggregated into major (left sub-plot), minor (middle sub-plot), and subset (right sub-plot) immune cell types. Minor cell types include five subtypes of T-cells, two subtypes of B-cells, and four subtypes of Myeloid. Subset cell types include eight subtypes of T-cells (four subtypes of CD4+ T cells and four subtypes of CD8+ T-cells), and nine subtypes of Myeloid (four subtypes of macrophages, two subtypes of monocyte, and three subtypes of DCs). Zero line indicates a perfect match between prediction and ground truth. Each box represents the middle 50% of raw prediction errors, which includes the first quartile (Q1), the median, and the third quartile (Q3). Upper and lower whiskers depict maxima and minima of raw prediction errors, excluding outliers. Outliers are raw prediction errors that are more than 1.5x the interquartile range from either Q1 or Q3. Source data are provided as a Source Data file.

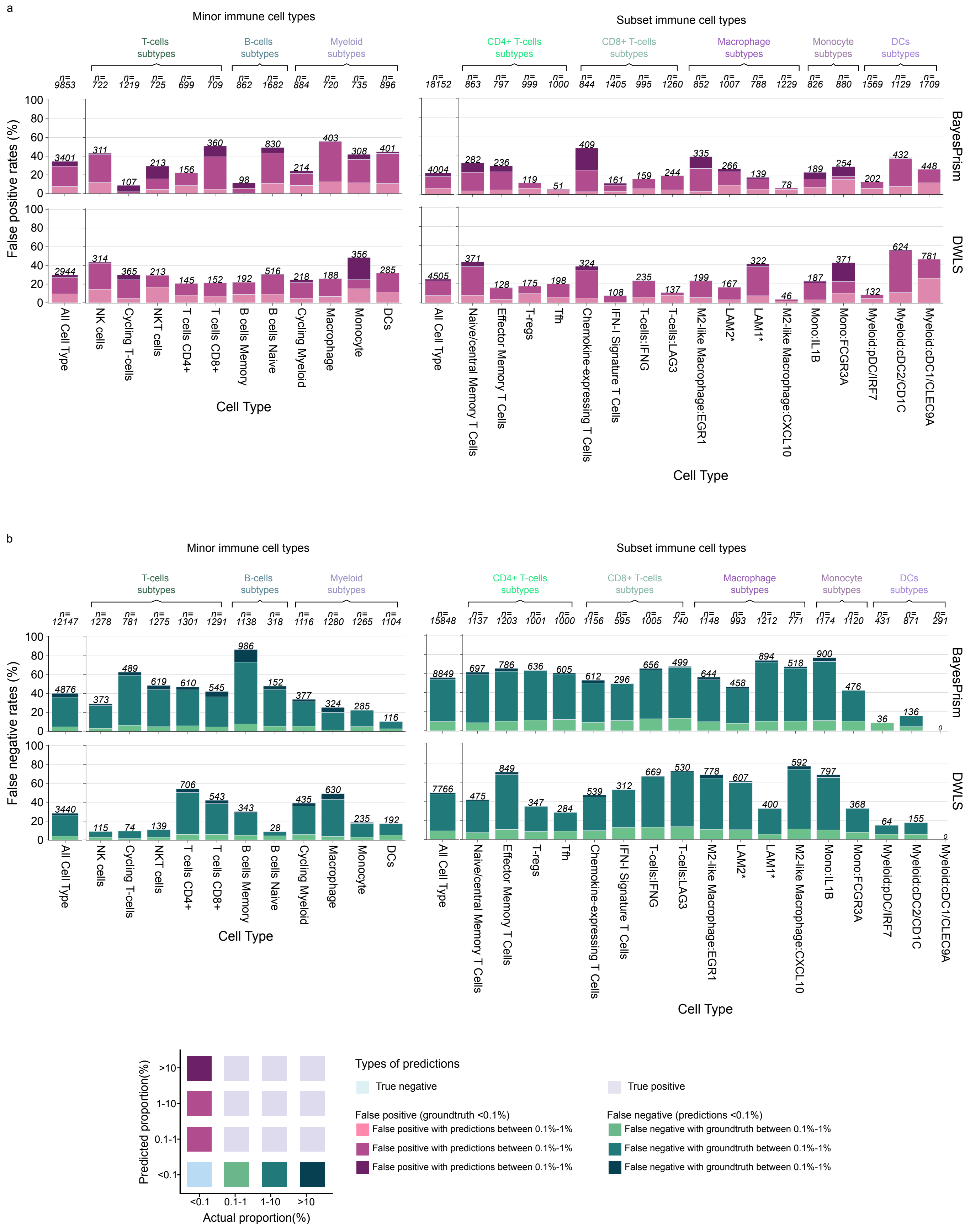
a
Minor immune cell types
Subset immune cell types
T-cells subtypes
B-cells subtypes
Myeloid subtypes
CD4+ T-cells subtypes
CD8+ T-cells subtypes
Macrophage subtypes
Monocyte subtypes
DCs subtypes
False positive rates (%)
BayesPrism
DWLS
All Cell Type
NK cells
Cycling T-cells
NKT cells
T cells CD4+
T cells CD8+
B cells Memory
B cells Naive
Cycling Myeloid
Macrophage
Monocyte
DCs
Naive/central Memory T Cells
Effector Memory T Cells
T-regs
Tfh
Chemokine-expressing T Cells
IFN-I Signature T Cells
T-cells:IFNG
T-cells:LAG3
M2-like Macrophage:EGR1
LAM2*
LAM1*
M2-like Macrophage:CXCL10
Mono:IL1B
Mono:FCGR3A
Myeloid:pDC/IRF7
Myeloid:cDC2/CD1C
Myeloid:cDC1/CLEC9A
Cell Type
b
False negative rates (%)
Predicted proportion(%)
Actual proportion(%)
Types of predictions
True negative
True positive
False positive (groundtruth <0.1%)
False positive with predictions between 0.1%-1%
False positive with predictions between 0.1%-1%
False positive with predictions between 0.1%-1%
False negative (predictions <0.1%)
False negative with groundtruth between 0.1%-1%
False negative with groundtruth between 0.1%-1%
False negative with groundtruth between 0.1%-1%

Supplementary Fig. 15: The performance of BayesPrism and DWLS methods for minor and subset immune cell types assessed by false positive and false negative rates. a) Percentages of the three levels of false positives out of the total number of false positives and true negatives across minor (left) and subset (right) immune cell types. Percentages and counts (shown above each bar) of false positives are either aggregated across all cell types (first stacked bar) or shown for each individual cell type. The total number of missing cell type components used to determine false positive rates for both methods is shown at the top with the "*n*=" prefix. **b)** Percentages of the three levels of false negatives out of total number of all false negatives and true positives across minor (left) and (subset) immune cell types. Percentages and counts (shown above each bar) of false negatives are either aggregated across all cell types (first stacked bar) or shown for each individual cell type. The total number of cell type components used to determine false negative rates for all methods is shown at the top with the "*n*=" prefix. Figure legend illustrates definitions of true negative, false positive, true positive, and false negative predictions. Source data are provided as a Source Data file.

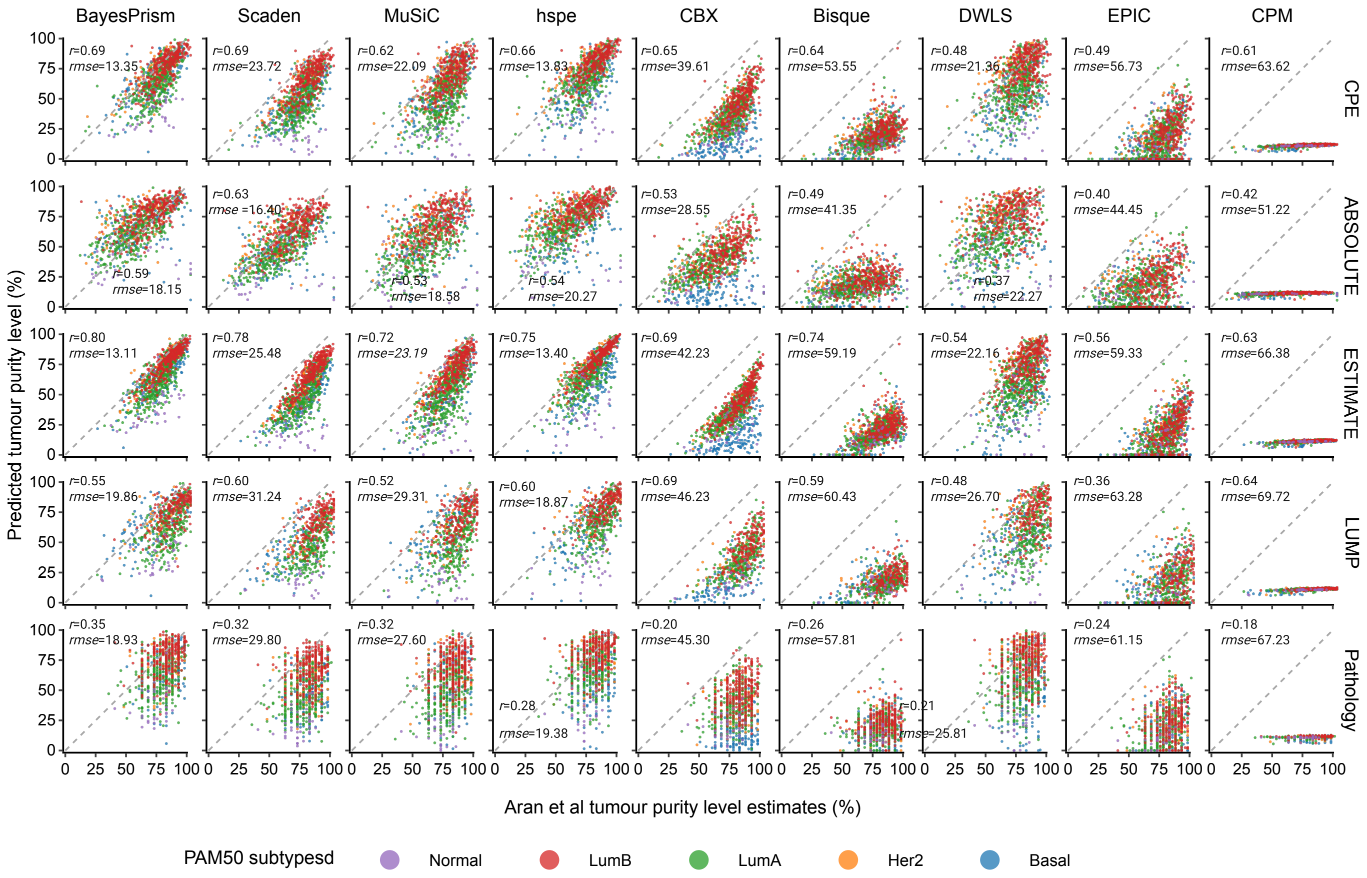

Supplementary Fig. 16: Comparison of nine deconvolution approaches to four alternative methods to predict tumour purity in TCGA breast data. Scatter plot comparing purity levels determined by the nine deconvolution methods (columns) with the four alternative methods (ABSOLUTE, ESTIMATE, LUMP and Pathology) and their Consensus Purity Estimates (CPE) provided by Aran et al. The data comprised *n=968* breast cancers from TCGA, with each sample coloured by the PAM50 subtype. The y-axis for each plot is the percentage of tumour cells predicted from the relevant deconvolution tool and the x-axis is the percentage of tumour cells predicted from the orthogonal method. Each scatterplot is annotated with the associated Pearson's r correlation coefficient and RMSE values. RMSE: Root Mean Square Error. Source data are provided as a Source Data file.

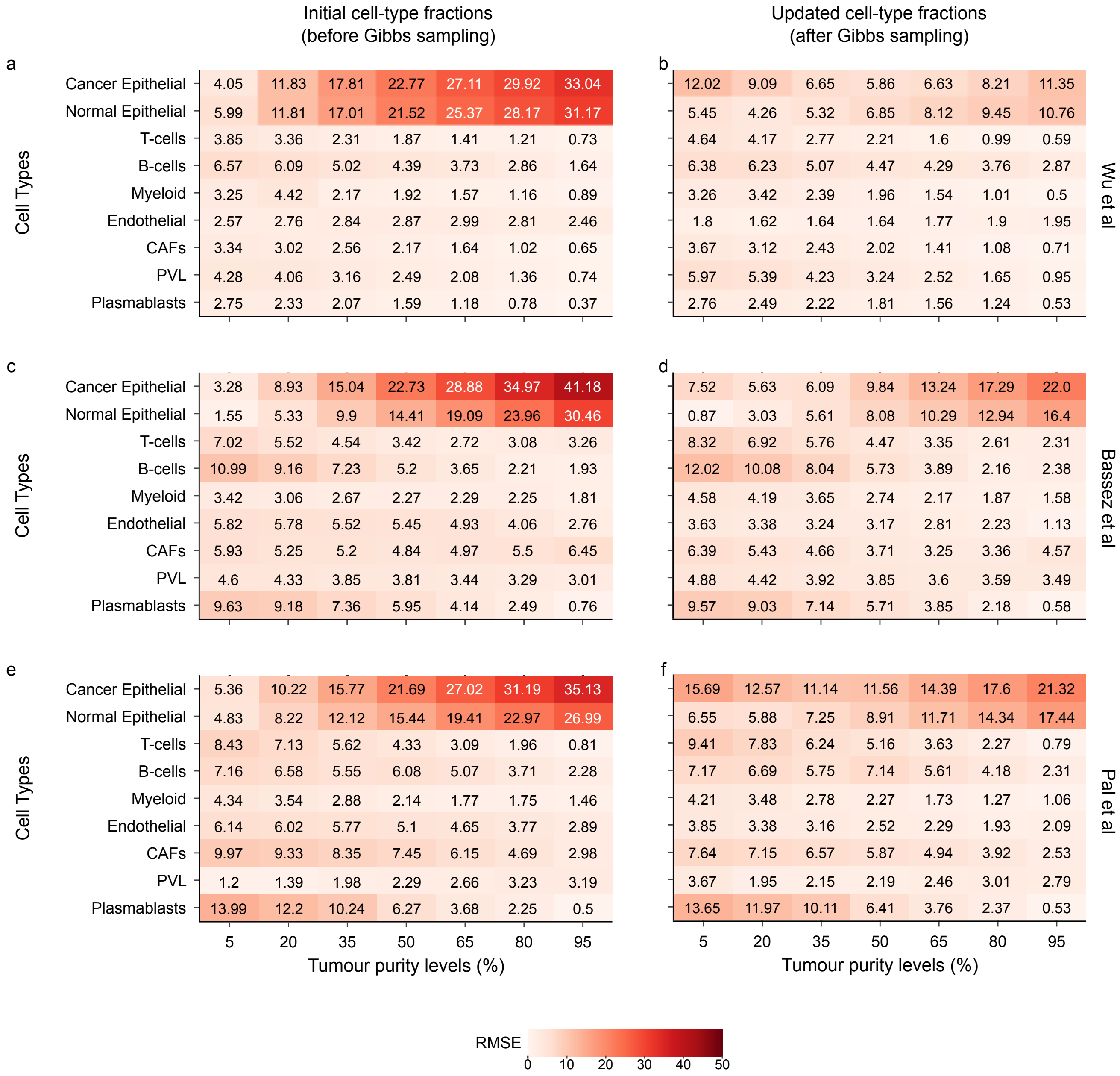

Supplementary Fig. 17: Performance of BayesPrism before and after Gibbs sampling on artificial bulk mixtures generated using scRNA-seq data from Wu et al, Bassez et al and Pal et al. RMSE between predicted and actual cell compositions of artificial bulk mixtures using scRNA-Seq from Wu et al **(a, b)**, Bassez et al **(c, d)** and Pal et al **(e, f)**, aggregated by cell type. Single-cell reference profiles and train simulated mixtures (only for Scaden) were from Wu et al, with intersecting genes with Bassez et al and Pal etl when appropriate. Left column **(a, c, e)** depict BayesPrism performance before Gibbs sampling, while right column **(b, d, f)** depict its performance after Gibbs sampling. Seven tumour purity levels are shown (from 5% to 95%, 15% interval). Darker shade of red represents higher RMSE values (worse performance), with numeric RSME values shown. CAFs: cancer-associated fibroblast, RMSE: Root Mean Square Error, scRNA-Seq: Single-cell RNA Sequencing. Source data are provided as a Source Data file.

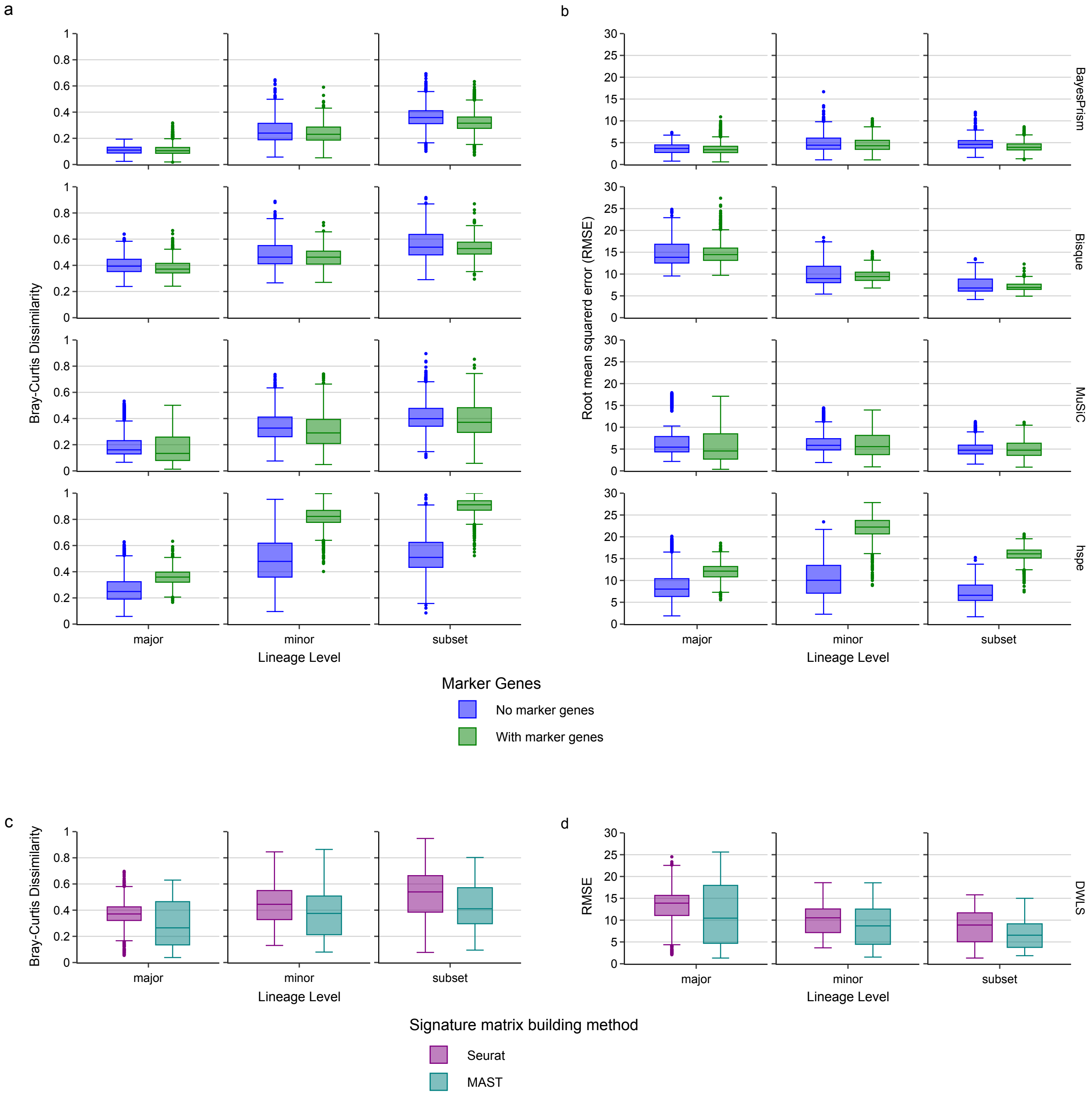

Supplementary Fig. 18: Performance comparison of different versions of BayesPrism, Bisque, MuSiC, hspe and DWLS. Bray Curtis dissimilarity **(a, c)** and Root-mean-squared errors (RMSE) **(b, d)** between predicted and ground truth cell compositions of BayesPrism, Bisque, MuSiC and hspe when marker genes are used (green) or not used (blue), and of DWLS when either Seurat (purple) or MAST (teal) is used for building its internal signature matrix. n=2,000 artificial bulk mixtures generated at 50% tumour purity and either major (top row), minor (middle row), or subset (bottom row) immune cell types. Each box represents the middle 50% of Bray-Curtis/RMSE values, which includes the first quartile (Q1), the median, and the third quartile (Q3). Upper and lower whiskers depict maxima and minima of Bray-Curtis/ RMSE values, excluding outliers. Outliers are Bray-Curtis/ RMSE values that are more than 1.5x the interquartile range from either Q1 or Q3. Higher Bray-Curtis dissimilarity and RMSE indicates poorer performance. Source data are provided as a Source Data file.

Supplementary references

1. Chu, T., Wang, Z., Pe’er, D. & Danko, C. G. Cell type and gene expression deconvolution with BayesPrism enables Bayesian integrative analysis across bulk and single-cell RNA sequencing in oncology. *Nat. Cancer* **3**, 505–517 (2022).
2. Casella, G. & George, E. I. Explaining the Gibbs Sampler. 9 (2022).
3. Jew, B. *et al.* Accurate estimation of cell composition in bulk expression through robust integration of single-cell information. *Nat. Commun.* **11**, 1971 (2020).
4. Newman, A. M. *et al.* Determining cell type abundance and expression from bulk tissues with digital cytometry. *Nat. Biotechnol.* **37**, 773–782 (2019).
5. Newman, A. M. *et al.* Robust enumeration of cell subsets from tissue expression profiles. *Nat. Methods* **12**, 453–457 (2015).
6. Frishberg, A. *et al.* Cell composition analysis of bulk genomics using single-cell data. *Nat. Methods* **16**, 327–332 (2019).
7. Tsoucas, D. *et al.* Accurate estimation of cell-type composition from gene expression data. *Nat. Commun.* **10**, 2975 (2019).
8. Racle, J., de Jonge, K., Baumgaertner, P., Speiser, D. E. & Gfeller, D. Simultaneous enumeration of cancer and immune cell types from bulk tumor gene expression data. *eLife* **6**, e26476 (2017).
9. Gregory J. Hunt & Johann A. Gagnon-Bartsch. The role of scale in the estimation of cell-type proportions. *Ann. Appl. Stat.* **15**, 270–286 (2021).
10. Hunt, G. J., Freytag, S., Bahlo, M. & Gagnon-Bartsch, J. A. dtangle: accurate and robust cell type deconvolution. *Bioinformatics* **35**, 2093–2099 (2019).
11. Cobos, F. A., Alquicira-Hernandez, J., Powell, J. E., Mestdagh, P. & Preter, K. D. Benchmarking of cell type deconvolution pipelines for transcriptomics data. *Nat. Commun.* **11**, 14 (2020).
12. Wang, X., Park, J., Susztak, K., Zhang, N. R. & Li, M. Bulk tissue cell type deconvolution with multi-subject single-cell expression reference. *Nat. Commun.* **10**, 380 (2019).
13. Menden, K. *et al.* Deep learning–based cell composition analysis from tissue expression profiles. *Sci. Adv.* **6**, (2020).
14. Wu, S. Z. *et al.* A single-cell and spatially resolved atlas of human breast cancers. *Nat. Genet.* **53**, 1334–1347 (2021).

15. Lambrechts, D. *et al.* Phenotype molding of stromal cells in the lung tumor microenvironment. *Nat. Med.* **24**, 1277–1289 (2018).

16. Aran, D., Sirota, M. & Butte, A. J. Systematic pan-cancer analysis of tumour purity. *Nat. Commun.* **6**, 8971 (2015).
