# Supplementary material for: Performance of tumour microenvironment deconvolution methods in breast cancer using single-cell simulated bulk mixtures
Source: Nat Commun. 2023 Sep 16;14:5758. doi: 10.1038/s41467-023-41385-5 (PMC10505141; doi:10.1038/s41467-023-41385-5)
Supplement: Supplementary file 3 — Description of Additional Supplementary Files [file 41467_2023_41385_MOESM3_ESM.pdf]

Description of Additional Supplementary Files

Supplementary Data 1. Summary of 26 patients and 100,064 single cells from Wu et al based on major, minor, subset cell types. The number of cells from each cell type in each patient from Wu et al. The samples are ordered by whether they were used in train or test and by their breast cancer molecular subtype: TNBC: triple negative breast cancer; HER2+: human epidermal growth factor receptor 2; ER+: estrogen receptor positive.

Supplementary Data 2. Total cell counts of each patient after data oversampling with SMOTE. The count of cells that were available for each cell type in the Wu et al samples after SMOTE. For each patient, cell types with counts < 10 were discarded and its count number was set to 0.

Supplementary Data 3. Median RMSE, and RPE of BayesPrism and DWLS across major, minor, and subset immune cell types. Assessment of the performance of BayesPrism and DWLS using Median RMSE and RPE. Median values were calculated over 2,000 mixtures for each cell type individually. RMSE: Root Mean Square Error; RPE: Relative Proportion Error.

Supplementary Data 4. Original genes list of each cohort in Bassez et al and their intersecting genes list. Cohort 1 are 29 patients who received only anti-PD1 treatment in Bassez et al. Cohort 2 are 11 patients who received neoadjuvant chemotherapy before anti-PD1 treatment in Bassez et al.

Supplementary Data 5. Intersecting genes between Wu et al and Bassez et al, and between Wu et al and Pal et al. Genes in each column are organised in alphabetical order. The order of genes in each column is independent of gene orders in other columns

Supplementary Data 6. Predicted tumour purity of each deconvolution method on bulk TCGA breast cancer mixtures, in comparison with ABSOLUTE-estimated tumour purity. Tumour purity estimations for the samples within the TCGA breast cancer dataset. Tumour purity estimations are shown for each deconvolution method, as well as other methods: ABSOLUTE-estimated, ESTIMATE-estimated, LUMP-estimated, Pathology-estimated and Consensus Purity Estimates (CPE).

Supplementary Data 7. Predicted lymphocyte population of each deconvolution method on bulk TCGA breast cancer mixtures, in comparison with Saltz et al estimated TILs population. The predicted lymphocyte population for the 9 deconvolution tools, which was compared to estimated TIL population by Saltz et al., was calculated as the total population of T cells and B cells. TILs: tumour-infiltrating lymphocytes.
