# Supplementary material for: Performance of tumour microenvironment deconvolution methods in breast cancer using single-cell simulated bulk mixtures
Source: Nat Commun. 2023 Sep 16;14:5758. doi: 10.1038/s41467-023-41385-5 (PMC10505141; doi:10.1038/s41467-023-41385-5)
Supplement: Supplementary file 11 — Reporting Summary [file 41467_2023_41385_MOESM11_ESM.pdf]

nature portfolio

Corresponding author(s): Nic Waddell and Khoa Tran

Last updated by author(s): Aug 15, 2023

Reporting Summary

Nature Portfolio wishes to improve the reproducibility of the work that we publish. This form provides structure for consistency and transparency in reporting. For further information on Nature Portfolio policies, see our Editorial Policies and the Editorial Policy Checklist.

Statistics

For all statistical analyses, confirm that the following items are present in the figure legend, table legend, main text, or Methods section.

n/a	Confirmed	
☐	☒	The exact sample size (*n*) for each experimental group/condition, given as a discrete number and unit of measurement
☐	☒	A statement on whether measurements were taken from distinct samples or whether the same sample was measured repeatedly
☐	☒	The statistical test(s) used AND whether they are one- or two-sided<br>*Only common tests should be described solely by name; describe more complex techniques in the Methods section.*
☐	☒	A description of all covariates tested
☒	☐	A description of any assumptions or corrections, such as tests of normality and adjustment for multiple comparisons
☐	☒	A full description of the statistical parameters including central tendency (e.g. means) or other basic estimates (e.g. regression coefficient) AND variation (e.g. standard deviation) or associated estimates of uncertainty (e.g. confidence intervals)
☒	☐	For null hypothesis testing, the test statistic (e.g. *F*, *t*, *r*) with confidence intervals, effect sizes, degrees of freedom and *P* value noted<br>*Give P values as exact values whenever suitable.*
☒	☐	For Bayesian analysis, information on the choice of priors and Markov chain Monte Carlo settings
☒	☐	For hierarchical and complex designs, identification of the appropriate level for tests and full reporting of outcomes
☒	☐	Estimates of effect sizes (e.g. Cohen's *d*, Pearson's *r*), indicating how they were calculated

Our web collection on statistics for biologists contains articles on many of the points above.

Software and code

Policy information about availability of computer code

Data collection	Publicly available datasets were downloaded from their sources using accession codes. This was done manually, and no software was used to download data.
Data analysis	This study uses previously published code of the deconvolution methods: BayesPrism (v2.0), bisqueRNA (v1.0.5), CIBERSORTx (available as of 29th June 2021), Cell Population Mapping (v0.1.6), DWLS (v0.1.0), EPIC (v1.1), hspe (v0.1), MuSiC (available as of 29th June 2021), Scaden (v1.1.2)<br><br>When data analysis was conducted in R, we used the packaged Seurat (v3.5). When data analysis was conducted in Python, we used the packaged Distance SMOTE (v0.4.0), pandas (v1.1.5), numpy (v1.19.15), scanpy (v1.7.2), scikit-learn (v0.24.2), and scikit-bio (v0.5.6). Data visualisation was done using the Python package plotly 5.15.0.<br><br>We developed custom code which is available at https://github.com/MedicalGenomicsLab/deconvolution_benchmarking

For manuscripts utilizing custom algorithms or software that are central to the research but not yet described in published literature, software must be made available to editors and reviewers. We strongly encourage code deposition in a community repository (e.g. GitHub). See the Nature Portfolio guidelines for submitting code & software for further information.

Data

Policy information about availability of data

All manuscripts must include a data availability statement. This statement should provide the following information, where applicable:

- Accession codes, unique identifiers, or web links for publicly available datasets
- A description of any restrictions on data availability
- For clinical datasets or third party data, please ensure that the statement adheres to our policy

This project used previously published scRNA-seq data available at GSE176078 [https://www.ncbi.nlm.nih.gov/geo/query/acc.cgi?acc=GSE176078] from Wu et al15, count data from [https://lambrechtslab.sites.vib.be/en/single-cell] for Bassez et al37 (raw data for this data is available at [https://ega-archive.org/studies/EGAS00001004809]), and GSE161529 [https://www.ncbi.nlm.nih.gov/geo/query/acc.cgi?acc=GSE161529] from Pal et al38. We accessed count level data that can be accessed publicly. RNA-seq data from TCGA for breast cancer was downloaded from UCSC Cancer Genomics Hub, currently available at [https://portal.gdc.cancer.gov]. Source data are provided with this paper.

Research involving human participants, their data, or biological material

Policy information about studies with human participants or human data. See also policy information about sex, gender (identity/presentation), and sexual orientation and race, ethnicity and racism.

Reporting on sex and gender	This study used publicly available data from breast cancers, and as such most data was from females. No sex- or gender-based analyses have been performed
Reporting on race, ethnicity, or other socially relevant groupings	Not applicable
Population characteristics	Not applicable
Recruitment	Not applicable
Ethics oversight	Not applicable

Note that full information on the approval of the study protocol must also be provided in the manuscript.

Field-specific reporting

Please select the one below that is the best fit for your research. If you are not sure, read the appropriate sections before making your selection.

☒ Life sciences ☐ Behavioural & social sciences ☐ Ecological, evolutionary & environmental sciences

For a reference copy of the document with all sections, see nature.com/documents/nr-reporting-summary-flat.pdf

Life sciences study design

All studies must disclose on these points even when the disclosure is negative.

Sample size	No sample size analysis was performed for this study. Each of he three breast cancer scRNA-Seq dataset (Wu et al, Bassez et al, and Pal et al) used to simulate artificial bulk mixtures in this study includes more than 100,000 cells, from 26 individuals in Wu et al, 42 individuals from Bassez and 27 individuals from Pal et al.<br><br>For each statistical analysis involving deconvolution performance on artificial bulk RNA-Seq mixtures, a minimum of 2,000 samples were simulated.<br><br>Each dataset was chosen due to their abundance of single cells (100,000+), and most patients in each datasets contain at least 5 major cell types each, which lends strength to the biological representativeness of the simulated pseudobulk mixtures. While most analyses were conducted using pseudobulks from Wu et al, the addition of Bassez et al and Pal et all was crucial in demonstrating the generalisability of the study's results.
Data exclusions	We excluded 8 individuals from Bassez et al and 3 individuals from Pal et al due to low cell counts. Additionally, Before performing Distance SMOTE and for each individual, only cell types with count>=10 were retained for Distance SMOTE and subsequent analysis.
Replication	The data oversampling step using SMOTE synthesizes new single cells randomly and cannot be reproduced. This data oversampling step was replicated on a patient-interdependent basis across datasets, i.e. synthesized cells for each patient were generated using only biological cells from the same patient.<br><br>In addition, performance of all deconvolution methods were assessed using scRNA-Seq data in Wu et al and validated with scRNA-Seq data from Bassez et al and Pal et al.

Randomization	We assigned 18 patients to training data and 8 patients to test data in Wu et al, 22 patients to training data and 12 patients to test data in Bassez et al, and 16 patients to training data and 8 patients to test data in Pal et al. This selection was performed manually to ensure all cell types are present in both training and test data. For the artificial bulk simulation process, only cells from one patient were randomly selected for each bulk mixtures, and record of the selected single cells was tracked using the unique single-cell identifiers.
Blinding	This study only used publicly availably datasets to simulate known bulk mixtures. For this reason, blinding was inapplicable.

Reporting for specific materials, systems and methods

We require information from authors about some types of materials, experimental systems and methods used in many studies. Here, indicate whether each material, system or method listed is relevant to your study. If you are not sure if a list item applies to your research, read the appropriate section before selecting a response.

Materials & experimental systems

n/a	Involved in the study
☒	☐ Antibodies
☒	☐ Eukaryotic cell lines
☒	☐ Palaeontology and archaeology
☒	☐ Animals and other organisms
☒	☐ Clinical data
☒	☐ Dual use research of concern
☒	☐ Plants

Methods

n/a	Involved in the study
☒	☐ ChIP-seq
☒	☐ Flow cytometry
☒	☐ MRI-based neuroimaging
